# Birch-Bark-Inspired Synergistic Fabrication of High-Performance Cellulosic Materials

**DOI:** 10.1021/acssusresmgt.4c00266

**Published:** 2024-11-13

**Authors:** Abdolrahim A. Rafi, Luca Deiana, Rana Alimohammadzadeh, Per Engstrand, Thomas Granfeldt, Staffan K. Nyström, Armando Cordova

**Affiliations:** † FSCN Research Center, Organic Chemistry, Mid Sweden University, Holmgatan 10, 851 70 Sundsvall, Sweden; ‡ FSCN Research Center, High Yield Pulp Technology, Mid Sweden University, Holmgatan 10, 851 70 Sundsvall, Sweden

**Keywords:** Cellulosic materials, Wet strength, Colloidal betulin particles, Hot-pressing, Eco-friendly, Water-based systems, Synergic interaction

## Abstract

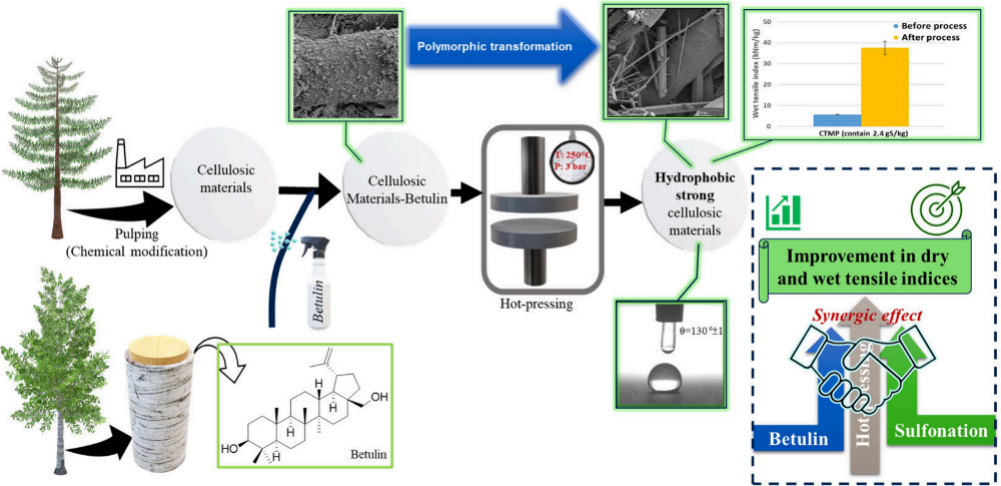

There is a growing demand for the utilization of sustainable materials, such as cellulose-based alternatives, over fossil-based materials. However, the inherent drawbacks of cellulosic materials, such as extremely low wet strength and resistance to moisture, need significant improvements. Moreover, several of the commercially available wet-strength chemicals and hydrophobic agents for cellulosic material treatment are toxic or fossil-based (e.g., epichlorohydrin and fluorocarbons). Herein, we present an eco-friendly, high-yield, industrially relevant, and scalable method inspired by birch bark for fabricating hydrophobic and strong cellulosic materials. This was accomplished by combining simple surface modification of cellulosic fibers in water using colloidal particles of betulin, an abundant triterpene extracted from birch bark, with sustainable chemical engineering (e.g., lignin modification and hot-pressing). This led to a transformative process that not only altered the morphology of the cellulosic materials into a more dense and compact structure but also made them hydrophobic (contact angles of up to >130°) with the betulin particles undergoing polymorphic transformations from prismatic crystals (betulin III) to orthorhombic whiskers (betulin I). Significant synergistic effects are observed, resulting in a remarkable increase in wet strength (>1400%) of the produced hydrophobic cellulosic materials.

## Abstract 2

This study presents a sustainable approach for dramatically enhancing the wet-strength and hydrophobicity of cellulosic materials by the synergistic combination of water-based betulin treatment, chemical modification, and hot-pressing.

## Introduction

Fossil-based plastics pose a significant global challenge, contributing to a staggering annual waste production of 400 million tons. With the projected global production of plastics expected to reach 1,100 million tons by 2050, there is an urgent demand to replace or minimize their use through the adoption of eco-friendly alternative materials.^1,2^ Cellulosic materials have emerged as excellent candidates for replacing or reducing the use of fossil-based counterparts, given their inherent advantages, such as biodegradability, biocompatibility, and sustainability. However, unmodified cellulosic materials often face challenges, including high wettability and low mechanical properties, especially under wet conditions.^3,4^ Enhancing these properties opens avenues for the further utilization of cellulose-based materials in diverse applications, including packaging,^5−7^ water repellent and self-cleaning materials,^8,9^ oil–water separation,^10,11^ water harvesting,^12^ and more.^13^ Numerous studies have been conducted to mitigate the drawbacks of pristine cellulosic materials. With respect to enhancing water repellency, common methods involve the use of low surface energy materials. For example, the incorporation of organofluorine compounds (“forever chemicals”) as hydrophobic agents.^9,14−16^ However, organofluorine compounds are expensive and known to pose environmental harm and pollution, raising serious health concerns due to their bio-accumulative nature.^17^ Non-fluorine-based compounds have also been utilized for this purpose. For instance, the use of various silanes^18−20^ or a combination of organocatalytic surface modification with organo-silanes, along with self-assembly or clays in the presence of a green catalyst, has resulted in improved hydrophobicity of nanocellulose films.^18−22^ Attachments of polymers, achieved through grafting to or from methods, also contribute to the enhancement of hydrophobicity in cellulosic materials. In this context, Hafrén and Córdova pioneered the use of organocatalysis on heterogeneous cellulose and paper for grafting of polyesters by ring-opening polymerizations or direct esterification of hydrophobic acids.^23^ Grafting of poly(lauryl acrylate) and poly(octadecyl acrylate) also produces hydrophobic cellulose fibers.^24^ An enzyme initiated reversible addition–fragmentation chain transfer (RAFT) polymerization followed by free-radical coupling between polymer chains and lignin was developed for the modification of jute fibers where poly butyl acrylate grafted into the fibers.^25^ Other methods such as esterification reactions,^26^ layer-by-layer techniques,^27^ etc.^28^ can also be used for achieving this purpose. Although promising, the recent common methods face several obstacles, such as the use of expensive and fossil-based chemicals (e.g., silanes^29^), multiple reaction and process steps, use of organic solvents, hard to scale, and/or being time consuming. These shortcomings call for the discovery of potential scalable methods that use ecological and biodegradable compounds. Substances provided by mother nature and originating from renewable natural resources can be a good solution. A great example is betulin (a natural triterpene) that constitutes 25–35 wt % of the outer white bark of birch trees.^30−33^ In addition, betulin is nontoxic and has health benefit properties, including anti-cancer properties.^31,34^ Rather than simply get incinerated together with the bark to produce thermal energy, betulin can be readily acquired by solvent extraction and be employed to fabricate value-added products such as hydrophobization agents.^30,32,35^ In this context, betulin and its derivatives can be utilized to produce hydrophobic cellulosic materials. Huang et al. demonstrated that the impregnation of cotton fabric with betulin or betulin terephthaloyl chloride copolymer resulted in hydrophobic surfaces with a contact angle of 151°.^36^ However, the method requires the use of organic solvents such as THF, toluene, and pyridine. Thus, there is a pressing need for developing betulin treatment in greener aqueous systems. Recently, Niu et al. demonstrated that a coating made of betulin, fossil-based polydimethylsiloxane, and a curing agent in 2-isopropanol can reach a high contact angle (149°) when applied on a cellulose substrate.^37^ Moriam et al. reported the fabrication of hydrophobic cellulosic materials using betulin and betulinic acid using ionic liquids (ILs) as solvents.^38^ The produced nonwovens and yarns samples (containing 10 wt % of betulin) reached water contact angles of approximately 100°. However, some ILs are considered toxic and hazardous materials and their residues can be entrapped inside the cellulosic products causing health and environmental issues.^39−42^ Moreover, ILs are not currently widely used for commercial purposes.

Alongside hydrophilicity, limitations in the mechanical strength of cellulosic materials, particularly in wet environments, present another major challenge when utilizing these materials.^43−48^ In the realm of mechanical strength enhancement for cellulosic materials, hot-pressing stands out as a recognized and effective method,^45−48^ yielding densified samples with improved mechanical properties. Moreover, hot-pressing is a convenient technique that has been successfully employed on an industrial scale for the manufacturing of commercial products. Delignified cellulose pulp (e.g. kraft pulp) exhibits tensile strength properties higher than those of lignocellulosic pulp.^43,44^ However, hot-pressing technology has been found to be highly beneficial and effective for lignocellulosic materials, which contain lignin, resulting in further improvement in mechanical properties.^45−48^ Moreover, the production of bleached pulps (e.g., kraft pulping) results in reduction of the pulping yield and involves the use of chorine-based bleaching agents that result in formation of toxic dioxins.^43^ Song et al.^45^ reported that partial delignification and subsequent hot-pressing of bulk wood samples resulted in lightweight materials with high strength and improved thermal stabilitie. The hot-pressing of lignin-containing samples, such as paper^46,47^ or wood,^48^ at temperatures above the *T*_g_ of lignin allows for a plastic-like flow of lignin and enhances the adhesion between fibers, where lignin and lignosulfonates act as natural binders. In this context, recent studies by the groups of Engstrand and Berglund have reported that sulfonation increases the softening and conformability at lower temperatures, resulting in higher strength.^49,50^

**Figure 1:**
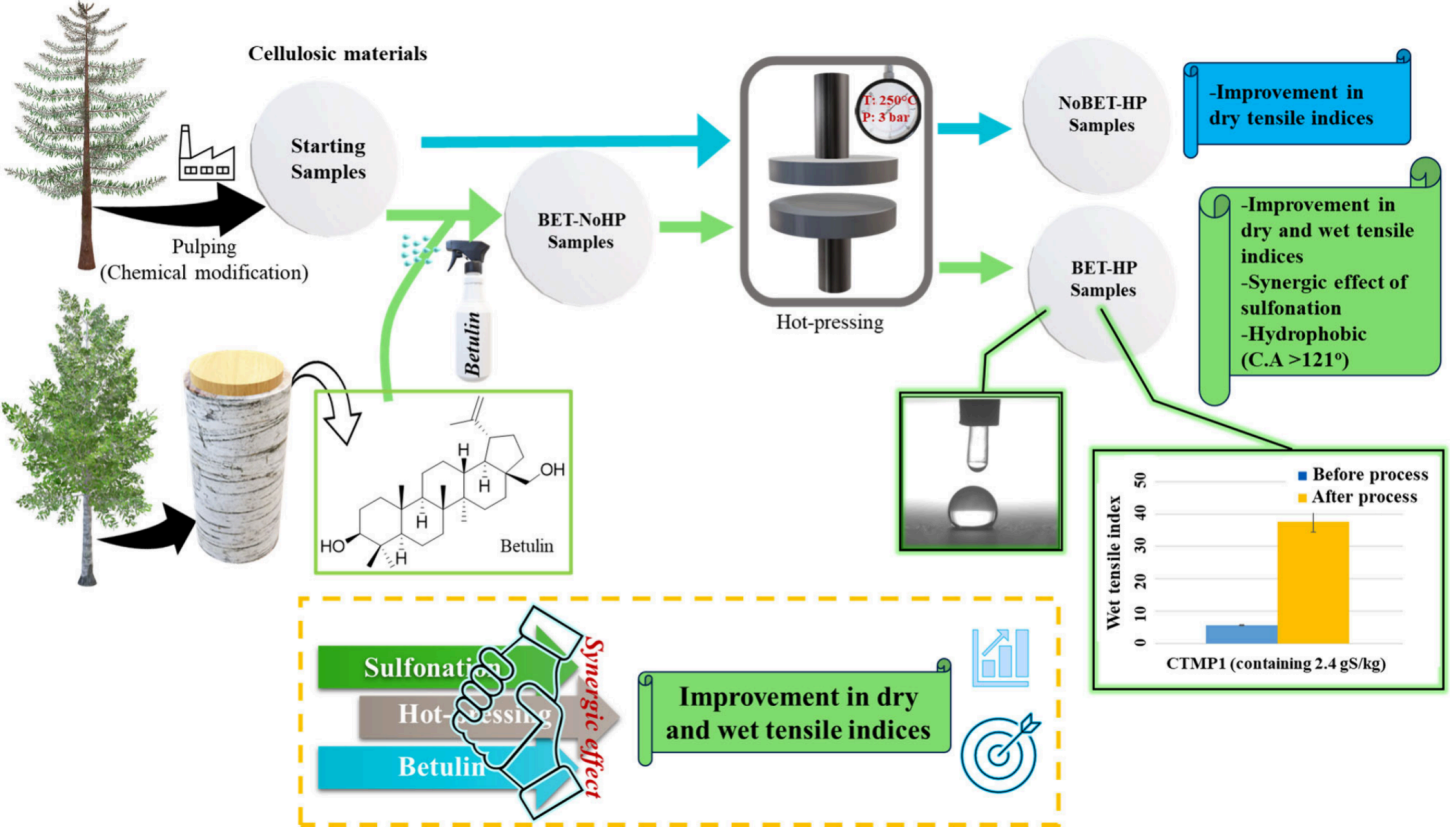
Schematic approach for preparation of the samples and their nomenclature. BET = Betulin, HP = Heat Pressing, and CTMP = Chemi-thermomechanical pulp.

Inspired by the assembly of birch-bark, we present a facile, sustainable, and scalable approach for fabricating hydrophobic and strong cellulosic materials by combining simple surface modification of cellulosic fibers with colloidal betulin particles in water and sustainable engineering [e.g., hot-pressing engineering and lignin modification during pulping (low-dose sulfonation, Figure 1)]. The disclosed industrially relevant process is performed in water-based systems and avoids the use of fossil-based chemicals or organic solvents. Moreover, the developed betulin/water formulation can readily be applied to a broad spectrum of cellulose materials and thereby make them hydrophobic (contact angles of up to >130°) and at the same time significantly increase their wet strength. The disclosed transformative sustainable process altered the morphology of the resulting hydrophobic cellulosic materials, which resulted in a dense and compact structure, while the betulin particles underwent polymorphic transformations from prismatic crystals (betulin III) to orthorhombic whiskers (betulin I). We observed a significant synergistic effect that led to increased wet strength and hydrophobicity of the fabricated cellulosic material. In fact, the wet strength of both unbleached and bleached fabricated hydrophobic cellulosic materials was dramatically increased (up to >1400%). In the case of the synergistic betulin/engineering treatment of pure cellulose filter paper, which initially had no measurable wet strength (0 kNm/kg), the resulting cellulose-betulin paper acquired a wet strength (4.9 kNm/kg), which was higher than most of the original CTMP samples (2–5.2 kNm/kg).

## Experimental Section

### Materials

Betulin (98%) was obtained from Holmen AB and Nature Science Technologies Ltd. Filter paper (No. 0B, diameter 18.5 cm, >99% cellulose) was obtained from Munktell. The raw material for pulping consisted of Norway spruce chips (Picea abies), of which 30% was sawmill chips, from Billerud Rockhammar mill. The pulp is from the mill trials in Rockhammar, 20–22 February 2019. The samples are all Chemi-ThermoMechanical Pulp (CTMP) with different amounts of bound sulfur (S). In the ChemiThermoMechanical pulping, the yield is around 95%, and the loss of material is proportional to the extractives, hemicellulose and lignin, in the starting wood chips. Thus, only a very small amount of lignin is removed. However, lignin is modified and more negative charges are introduced to the pulp by alkaline ester hydrolysis and sulfonation by the added sulfur. CTMP1 (total bound S of 2.4 g(S)/kg) and CTMP2 (total bound S of 1.0 g(S)/kg) with 28% dryness are from the primary high-consistency refiner. CTMP3 with 85% dryness is from the final step and the same as CTMP2 (total bound S of 1.0 g(S)/kg). Pulp preparation and additional information can be found in the Supporting Information.^51^ The Klason lignin content of CTMP1 is 27.0%, of CTMP2 is 27.0%, and of CTMP3 is 26.9% (TAPPI T222 analysis). The acid soluble lignin of CTMP1 is 0.7%, that of CTMP2 is 0.6%, and that of CTMP3 is 0.5% (TAPPI T-UM 250 analysis).

#### Procedure for Making Handmade Paper Sheets of CTMP

The handmade laboratory CTMP paper sheets were made according to the ISO 5269-2 method using a Rapid-Köthen sheet former instrument. CTMP (50 g dry mass) was dissolved in 2 L of water, stirred for 15 min, and finally disintegrated with a disintegration machine. Afterwards, the mixture was diluted with water to around 8 kg total weight and stirred for 10 min. Next, the mixture was transferred into the Rapid-Köthen sheet former, and the resulting wet CTMP hand sheets were dried at 95°C under pressure of 96 kPa for 10 min.

#### Preparation of Colloidal Particle Betulin Water Suspension

Deionized water (300 mL) was slowly added to betulin (6 g, 13,55 mmol) and vigorously stirred at room temperature for 1 h. Next, the suspension was sonicated at 35°C for 1.5 h to give a homogeneous milky solution. The particle size is 857 ± 16 nm, and the polydispersity index is 0.31 ± 0.06 as determined by a dynamic light scattering instrument (ZetasizerNano-ZSP, Malvern Instruments). Before measurement, the prepared betulin/water suspension was diluted to 200 μg/mL, and the measurement was performed in triplicate at 25°C.

#### Preparation of Betulin No Hot-Pressing (BET-NoHP) and Betulin Hot Pressing (BET-HP) Samples

The prepared CTMP handmade sheets and filter paper samples were sprayed with the prepared betulin suspension and successively dried in an oven at 55 °C for 1 h, giving no hot-pressing (BET-NoHP) samples. Finally, samples were hot-pressed at 3 bar and 250 °C for 1 min, giving betulin hot-pressed (BET-HP) samples. The hot-pressing times of 30 s and 2 min gave similar results as 1 min hot-pressing.

## Results and Disscusion

We began developing different betulin formulations for the treatment of cellulosic materials. Initially we prepared various formulations of betulin in ethanol (Table S1). We found that the water contact angles (WCAs) of the different cellulose substrates were improved. However, they never reached levels higher than 90°. To our delight, we were able to develop an aqueous colloidal particle formulation of betulin that after impregnating various cellulosic samples improved their WCAs (up to 130°, Table S2). With this colloidal particle water suspension of betulin (particle size of around 857 ± 16 nm and polydispersity index of 0.31 ± 0.06) in hand, we were ready to investigate a birch-bark inspired approach for fabricating strong and hydrophobic cellulosic materials by combining industrially relevant chemical modification, sustainable betulin treatment, and hot-pressing (Figure 1).

**Figure 2:**
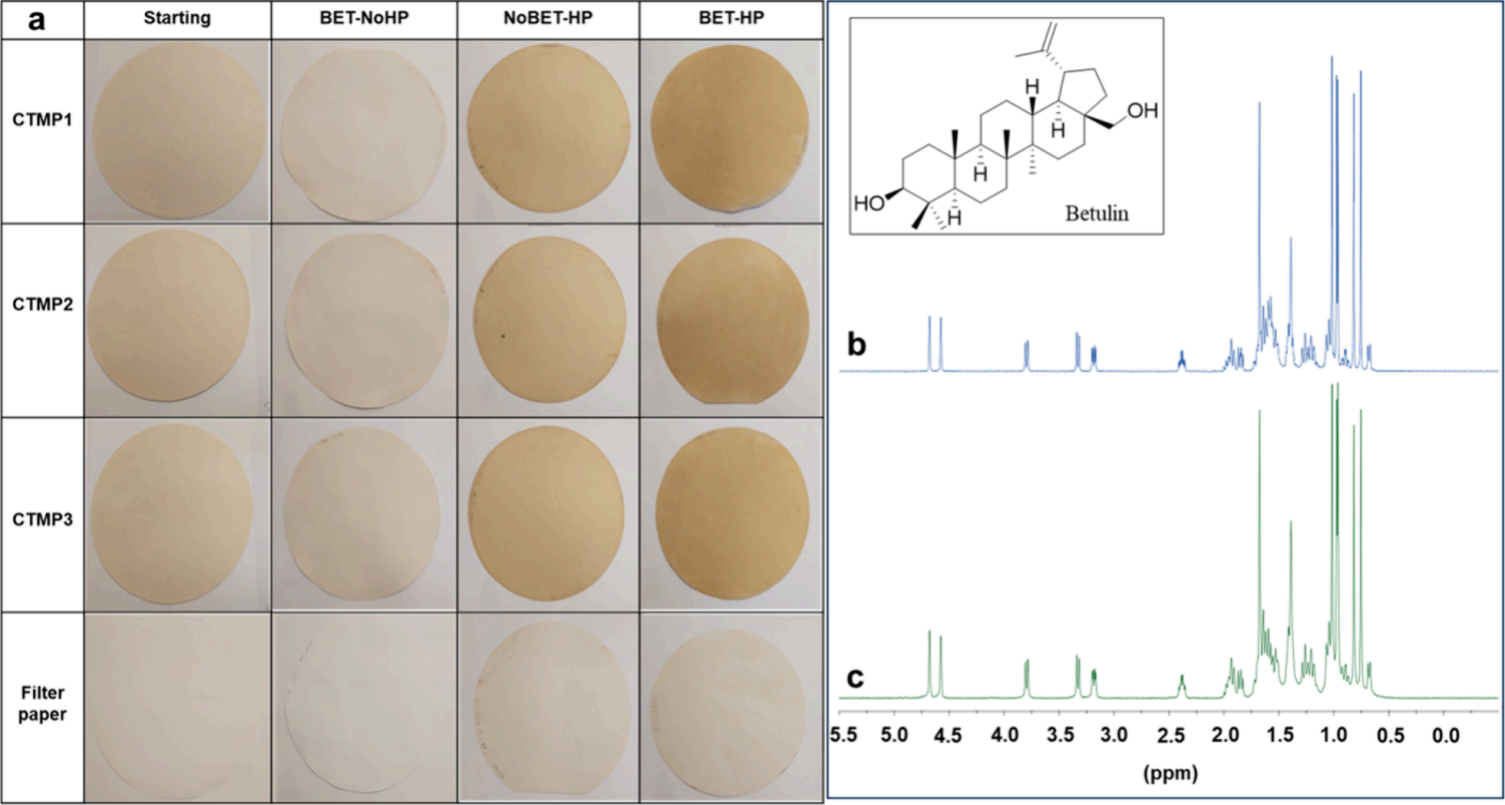
(a) Images of filter paper and all CTMP samples without betulin and without hot-pressing (starting sample), with betulin without hot-pressing (BET-NoHP), with betulin without hot-pressing (NoBET-HP), and with betulin and with hot-pressing (BET-HP). (b) ^1^H-NMR spectra of betulin and (c) ^1^H-NMR spectra of betulin taken from the BET-HP sample (hot-pressing at 280°C).

We simply treated the pristine and untreated cellulosic samples (i.e., starting samples) with the colloidal betulin particle/water suspension by spraying, which resulted in the corresponding BET-NoHP samples (Figure 1). Afterwards, the betulin-treated cellulosic (BET-NoHP) samples were hot-pressed at 3 bar, 250°C, and for 1 min to produce the corresponding betulin-hot-pressed (BET-HP) samples. As can be seen in Figure 2, the BET-NoHP samples have a whiter color because of the betulin (BET) applied at the surface of the paper sheets (∼6 wt % of betulin, Table S3). The nuclear magnetic resonance (NMR) spectra of betulin were identical with the sample scraped from the surface of the BET-HP samples (Figure 2b,c). NMR analyses of betulin, which had been melted at 280°C, were also identical. Thus, betulin was stable during the hot-pressing process. This was also supported by the IR-analyses. However, a change in the morphology of the betulin crystals was observed as described in the scanning electron microscopy (SEM) and X-ray diffraction (XRD) analysis sections.

### Fourier Transform Infrared (FT-IR) Spectra

**Figure 3:**
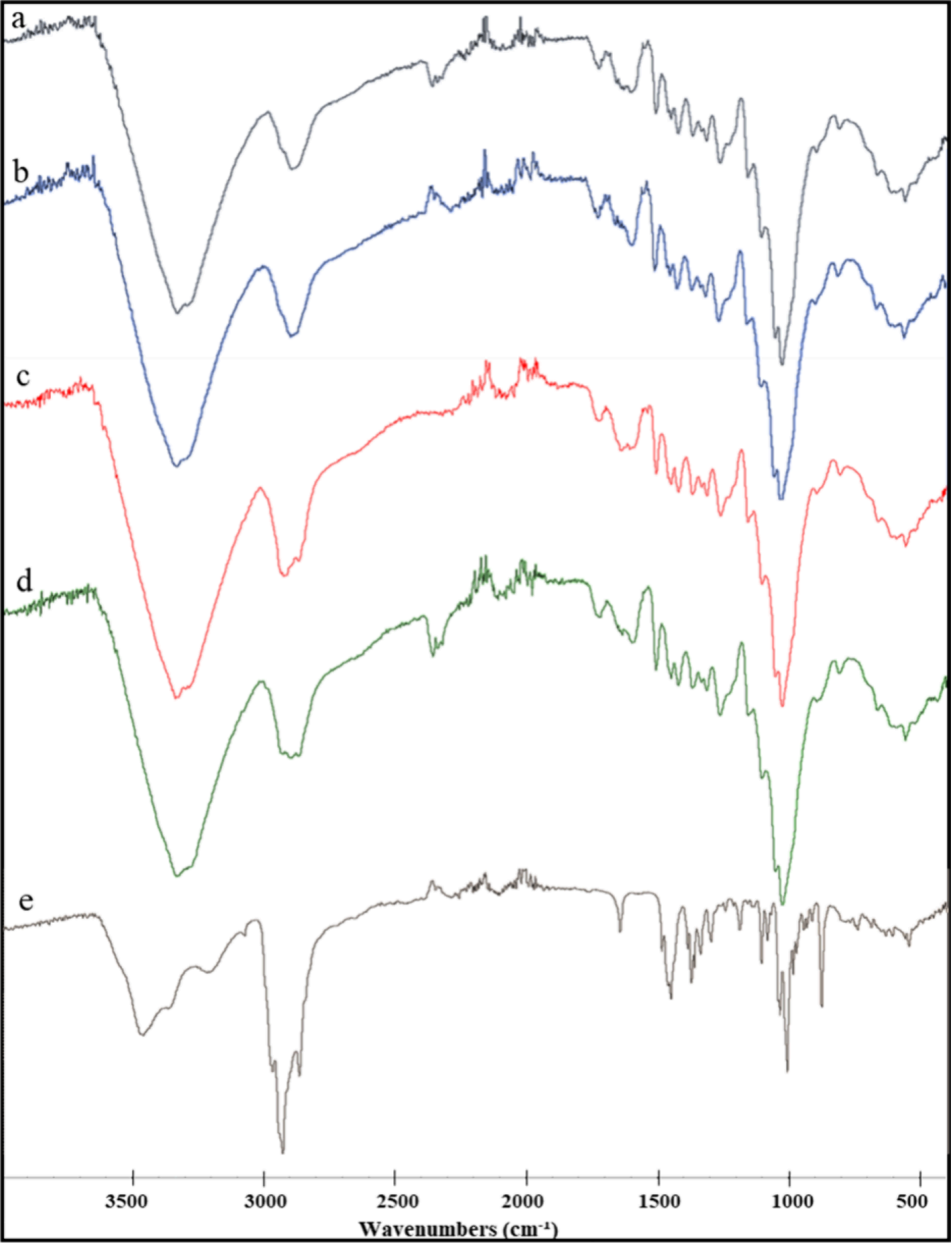
FTIR spectra of CTMP1-starting (a), CTMP1-NoBET-HP (b), CTMP1-BET-NoHP (c), CTMP1-BET-HP (d), and pure betulin (e).

The FT-IR spectra of CTMP1-starting, CTMP1-NoBET-HP, CTMP1-BET-NoHP, and CTMP1-BET-HP are shown in Figure 3. FT-IR spectra of the other prepared samples were also recorded and are shown in Figures S7–S9. In this section, the IR analyses of the CTMP1 samples are shown and discussed since all other CTMP samples also showed similar FT-IR spectra. In Figure 3, the wide absorption peaks in all spectra around 3331 and 2895 cm^–1^ correspond to the O–H and C–H stretching vibrations, respectively. The absorption bands around 1631 cm^–1^ belong to the bending vibrations of the hydroxyl groups of absorbed water (Figure 3). The peaks for hemicellulose (C=O stretching) and lignin (aromatic ring skeleton vibration) are around 1730 and 1509 cm^–1^, respectively.^50^ The absorption bands around 1158 and 1105 cm^–1^ are attributed to the stretching vibrations of C–C and C–O, respectively. The absorption peaks around 1028 cm^–1^ come from the C–O–C vibration in the pyranose.^52^ Furthermore, in the case of spectra of the samples containing betulin (i.e., spectra c and d), new peaks appeared around 2895 cm^–1^ which originate from betulin (aliphatic C–H stretching). Similar FT-IR spectra were observed for the other samples as well (Figures S7–S9).

### Scanning Electron Microscopy (SEM)

**Figure 4:**
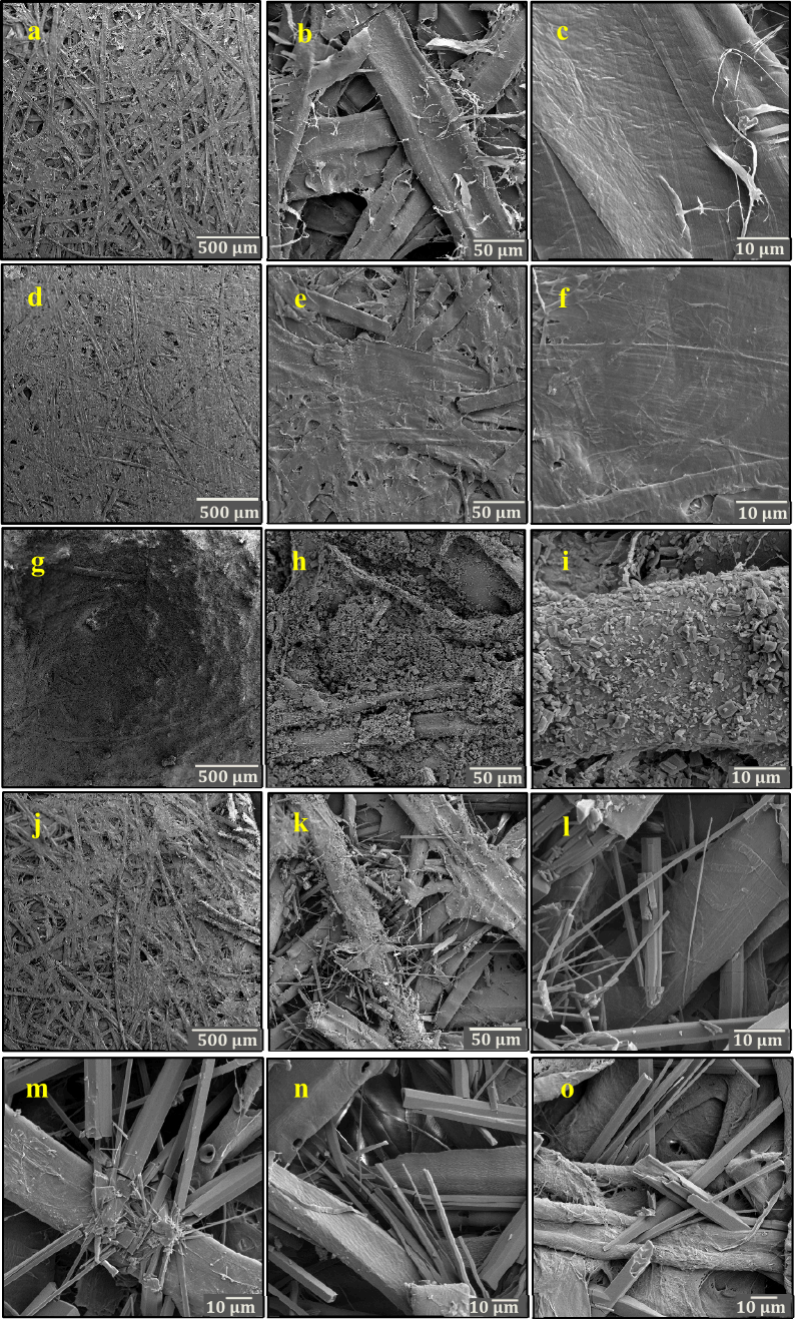
SEM images of CTMP1-starting (a–c), CTMP1-NoBET-HP (d–f), CTMP1-BET-NoHP (g–i), CTMP1-BET-HP (j–l), CTMP2-BET-HP (m), CTMP3-BET-HP (n), and filter paper-BET-HP (o).

Figure 4 depicts the SEM images of the starting CTMP1 (CTMP1-starting), CTMP1-NoBET-HP, CTMP1-BET-NoHP, CTMP1-BET-HP, as well as CTMP2-BET-HP, CTMP3-BET-HP, and filter paper-BET-HP samples. The surface SEM image of CTMP1-starting reveals numerous cellulose macrofibers intertwined together, forming a complex network (Figure 4a). Comparing pictures of CTMP1-starting (Figure 4a–c) with those of CTMP1-NoBET-HP (Figure 4d–f) shows that hot-pressing has changed the surface morphology of the sample, and as a result a compact and dense structure has been obtained. After spraying the CTMP1-starting sheet with the colloidal betulin aqueous suspension, it can be seen that the surfaces of the fibers of the resulting CTMP1-BET-NoHP sample are covered by the betulin particles (Figure 4g–i). The particles had a prismatic shape of previously reported betulin hydrate (betulin III).^53,54^ The SEM images of CTMP1-BET-HP (j–l) show the polymorphic transformation of betulin from prismatic crystals (betulin III) to long whiskers (betulin I).^54^ Betulin I whiskers are also observed in the SEM images of the CTMP2-BET-HP (Figure 4m), CTMP3-BET-HP (Figure 4n), and filter paper-BET-HP samples (Figure 4o). In fact, SEM images starting from CTMP2, CTMP3, and filter paper, respectively, revealed that all samples treated by the betulin water suspension/hot-pressing technology underwent the same structural morphology changes (Figures S10–S12) as shown for CTMP1 to finally form long betulin I whiskers intertwined with the cellulosic fibers (Figure 4m–o). We also immersed the samples CTMP1-starting, CTMP1-NoBET-HP, CTMP1-BET-NoHP, and CTMP1-BET-HP in water for 1 min followed by drying. SEM analysis of these samples (Figure S13) showed a morphology similar to the corresponding dry samples, with the structure of the CTMP1-BET-HP sample being least affected by immersion in water.

**Figure 5:**
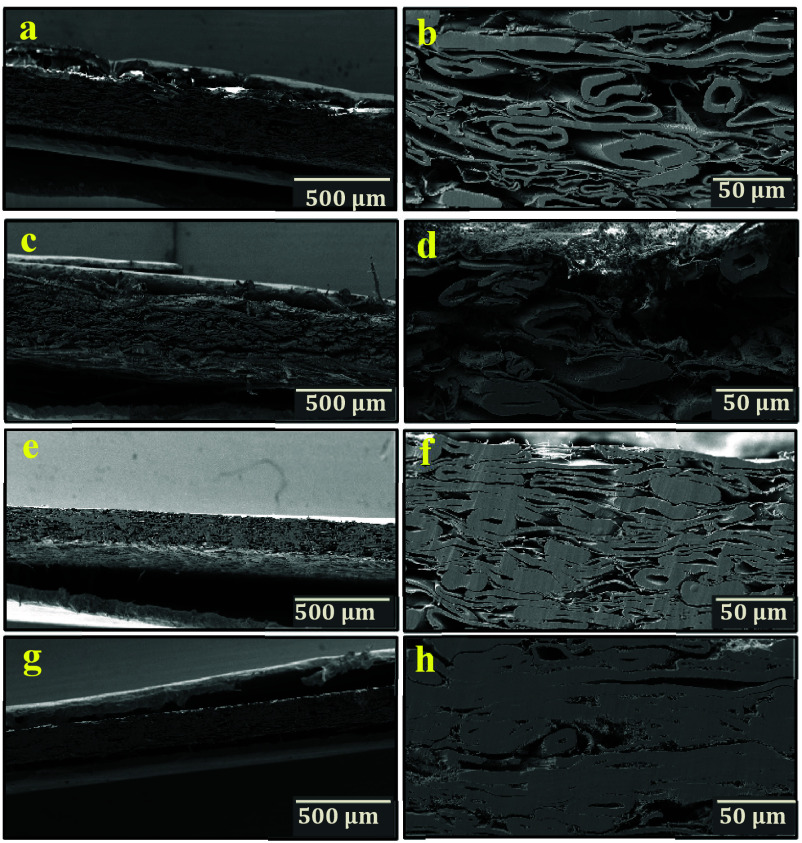
Cross-sectional SEM images of CTMP1-starting (a, b), CTMP1-BET-NoHP (c, d), CTMP1-NoBET-HP (e, f), and CTMP1-BET-HP (g, h).

Figure 5 depicts the cross-sectional images of CTMP1-starting, CTMP1-BET-NoHP, CTMP1-NoBET-HP, and CTMP1-BET-HP, respectively. The cross-sectional SEM image of sample CTMP1-starting shows a loosely packed structure with some interfiber pores and open fiber lumens (Figure 5a,b). The cross-sectional SEM image of the CTMP1-BET-NoHP sample (Figure 5c,d) reveals that the betulin particles are mostly placed on the surface of the sheet. In comparison to the CTMP1-starting sample, the CTMP1-BET-NoHP sample exhibits a looser structure with larger pores and as a result has a thicker cross section. After hot-pressing, the fiber pores mostly disappeared, the thickness decreases, and a densely packed structure is formed (Figure 5g,h).

### X-ray Diffraction (XRD) Analysis

**Figure 6:**
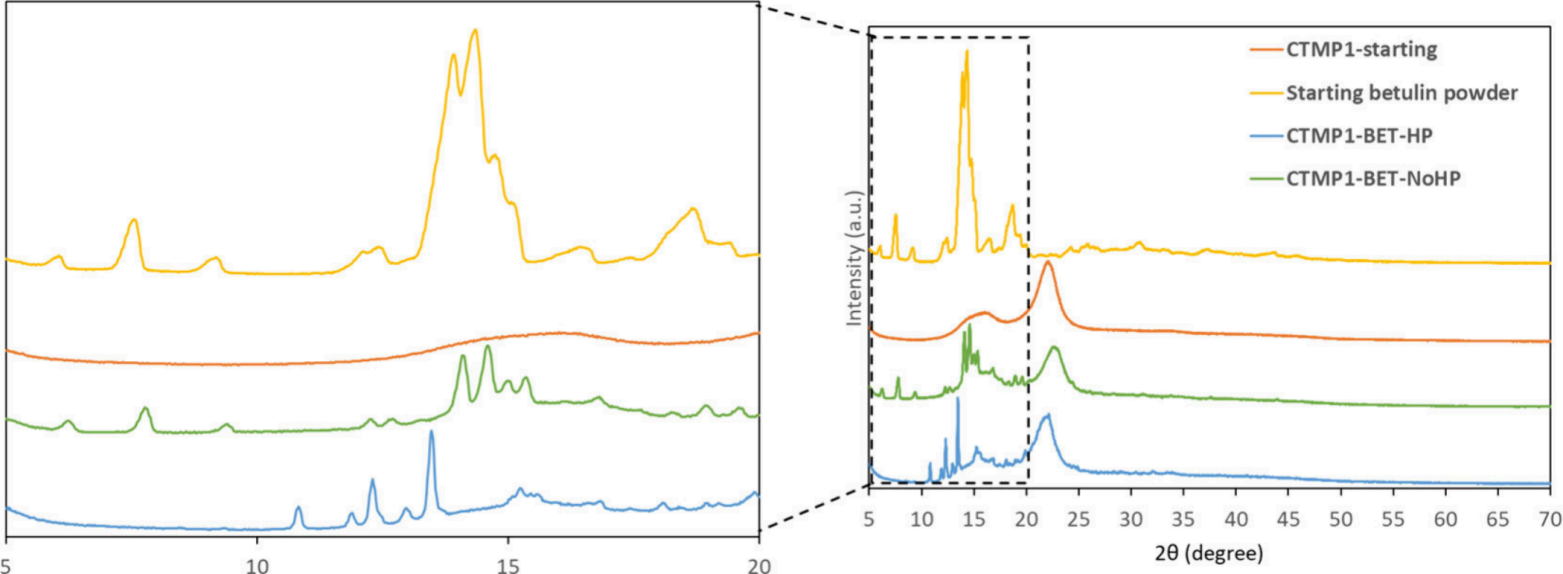
XRD spectra of starting pure betulin powder, CTMP1-starting, CTMP1-BET-NoHP, and CTMP1-BET-HP.

Betulin can undergo polymorphic changes and form different types of crystal structures.^53,54^ For example, it can form prismatic crystals of betulin hydrate (betulin III) and long whiskers (betulin I). These types of crystal structures can indeed be seen in the SEM images of the betulin treated sheets (Figure 4). The XRD patterns of starting betulin powder, CTMP1-starting, CTMP1-BET-NoHP, and CTMP1-BET-HP are depicted in Figure 6. The starting betulin powder spectrum shows several distinct peaks that correspond to the prismatic crystals of betulin hydrate (betulin III)^54^ at the diffraction angles of 6.05°, 7.55°, 9.19°, 12.12°, 12.43°, 13.92°, 14.35°, 14.73°, 15.08°, 16.43°, 18.66°, 19.34°, 19.95°, 24.28°, 25.88°, 30.86°, 37.40°, and 43.63°. The CTMP1-BET-NoHP spectrum as compared with the untreated CTMP1-starting spectrum shows the appearance of new peaks at 6.26°, 7.77°, 9.39°, 12.27°, 12.71°, 14.09°, 14.61°, 15.00°, 15.36°, 16.82°, 18.94°, 19.59°, 20.36°, and 24.39°, which corresponds to the formation of the betulin III prismatic structure. The spectrum of the hot-pressed sample CTMP1-BET-HP reveals that a polymorphic transformation has occurred to form orthorhombic whiskers (betulin I)^54^ with new peaks appearing at 10.83°, 11.88°, 12.31°, 12.97°, 13.48°, 15.24°, 15.56°, 15.44°, 16.84°, 18.07°, 18.96°, 19.91°, and 24.87°. Thus, the staring prismatic crystals of the starting betulin form III were converted to whiskers at 250 °C following subsequent water removal and the III → I polymorphic transformation. These changes are also in accordance with what the SEM images of these samples revealed (Figure 4).

### Water Contact Angle (WCA)

WCA measurements are a quantitative assessment of the wettability of a solid surface by water. A surface is typically considered hydrophobic if it has a WCA of 90° or greater. In Table 1, the WCAs of different samples after 5 min are shown. A clear synergistic effect was observed for the combined betulin treatment/hot-pressing samples with WCAs reaching 132° (entries 1–4). The measured WCAs for the starting untreated reference samples (entries 5–8) were 0°. In fact, the applied water drops during the measurements began to adsorb immediately into the surface of the samples. In addition, the WCAs for the hot-pressed samples without betulin treatment were 0° (entries 9–12). The spraying of colloidal betulin particle/water suspension onto the surface of filter paper without hot-pressing did not improve the WCA (entry 13). Hot-pressing betulin-treated filter paper for a longer time at a low temperature (97°C) was also not effective (entry 14). Thus, there is a clear synergistic effect for achieving a hydrophobic surface when betulin treatment by spraying is combined with hot-pressing. In addition, we noticed that impregnating the filter paper with the betulin/water suspension instead of spraying gave a hydrophobic surface and a WCA of 110° (Table S2). However, even soaking the filter paper with betulin gave a lower value than combined aqueous betulin spraying and hot-pressing treatment. Thus, the polymorphic transformation of betulin from prismatic crystals (betulin III) to long whiskers (betulin I) significantly improves the hydrophobicity of the cellulosic substrates.

**Table 1:** WCA Measurements Taken for Different Samples

Entry	Cellulosic material	WCAs (θ)^a^
1^b^	Filter paper-BET-HP	121 ± 2°
2^b^	CTMP3-BET-HP	132 ± 1°
3^b^	CTMP2-BET-HP	121± 2°
4^b^	CTMP1-BET-HP	130 ± 1°
5	Filter paper-starting	0°
6	CTMP3-starting	0°
7	CTMP2-starting	0°
8	CTMP1-starting	0°
9^b^	Filter paper-NoBET-HP	0°
10^b^	CTMP3-NoBET-HP	0°
11^b^	CTMP2-NoBET-HP	0°
12^b^	CTMP1-NoBET-HP	0°
13	Filter paper-BET-NoHP	0°
14^c^	Filter paper-BET-HP	0°

a The WCAs were measured after 5 min.

b BET-HP and NoBET-HP samples are hot-pressed at 250 °C, 3 bars for 1 min.

c The sample pressed for 20 min at 1 bar and 97 °C.

### Tensile Strength

**Figure 7:**
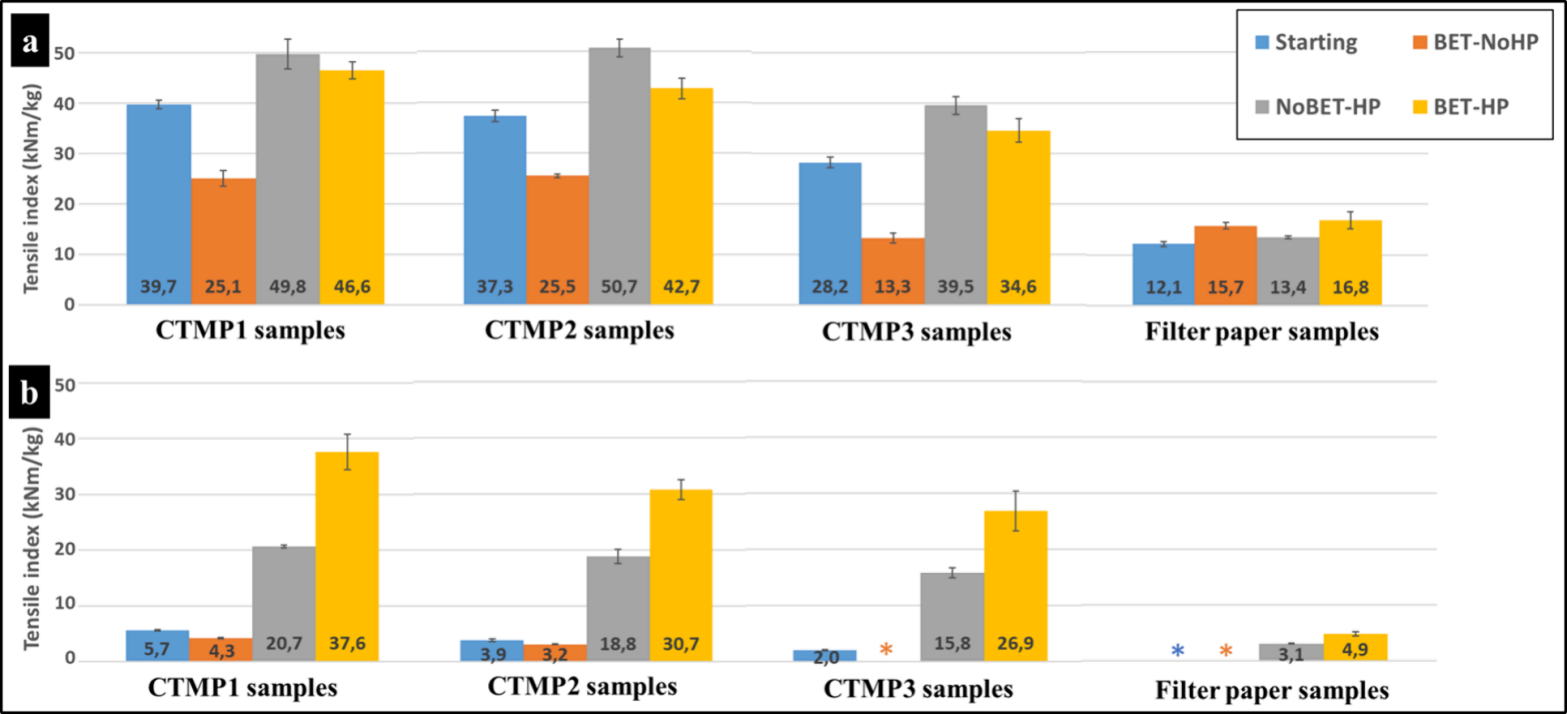
(a) Dry tensile indices and (b) wet tensile indices of the samples.

Figure 7a depicts the tensile indices of the samples in the dry state. The dry tensile index of CTMP1-starting (blue bar) is higher than that of for CTMP2-starting and CTMP3-starting, respectively. This is due to a higher degree of sulfonation (i.e., higher amount of S) in CTMP1.^49,55^ The tensile indices of CTMP-starting samples decrease after spraying with the colloidal betulin particle/water suspension (Figure 7a, BET-NoHP samples). This is in accordance with previous observation that the addition of betulin can reduce the strength of cellulose materials by 10–20%.^56^ This was attributed to the presence of additives that can interfere with the structure and network of cellulosic materials creating discontinuities in the cellulose structure, which results in lower strength.^38,57^ Hot-pressing noticeably improves the tensile indices of the starting dry samples due to densification.^47^ However, the most dramatic increase in dry tensile indices can be seen when hot-pressing is performed on the cellulosic samples treated with the colloidal betulin particle/water suspension (Figure 7a, orange versus yellow bars). When comparing the tensile indices of the starting cellulosic samples (blue bars) with those of the BET-HP samples (yellow bars), a clear and significant improvement in dry strength can be observed for all samples through our sustainable process. In the case of filter paper (Figure 7a), the spraying of the colloidal betulin particle/water suspension increases the dry tensile index of the pure cellulose paper by up to 30%. Figure 7b shows the wet tensile indices of the fabricated cellulose-based sheets. All the initial cellulose-based sheets (CTMP1, CTMP2, CTMP3, and filter paper) exhibited very low wet strength, with filter paper demonstrating the lowest wet tensile strength (<0.01 N/m). Spraying the aqueous colloidal particle betulin suspension onto the various cellulose-based sheets further reduced their wet strength. For example, the fabricated CTMP3-BET-NoHP sheet had a wet tensile strength of <0.01 N/m. It is noteworthy that the hot-pressing significantly increased the wet strength. For instance, hot-pressing the CTMP sheets improved the wet strength (tensile indices of up to 21 kNm/kg, Figure 7b). This can be explained by noting that upon hot-pressing at temperatures above lignin *T*_g_ the lignin of these samples softens and allows for a plastic like flow. Hence, the softened lignin facilitates robust inter-fiber bonding, effectively adhering fibers together; in essence, lignin serves as a natural wet-strength additive.^45−47^ Furthermore, the wet tensile index of the CTMP1-NoBET-HP sample was slightly higher than that of CTMP2-NoBET-HP (∼10%), which can be attributed to the greater amount of sulfur (i.e., higher sulfonation) present in CTMP1-NoBET-HP. Higher sulfonation leads to more charges and lowers the lignin *T*_g_. This makes the hot-pressing more efficient and results in higher strengths in the samples.^47,48^ The wet tensile strengths of filter paper-starting and filter paper-BET-NoHP samples as well as CTMP3-BET-NoHP were less than 0.01 N/m (* in Figure 7b). Indeed, all samples treated with colloidal betulin particle/water suspension experienced a decrease in wet strength. However, a remarkable synergistic increase in wet strength (>1400%) was observed in the CTMP samples when hot-pressing was combined with aqueous betulin surface engineering and integrated chemical modification during pulping (i.e., low dose sulfonation; Figure 1). For example, the wet strength index of CTMP3 increases from 2.0 to 26.9 kNm/kg by the synergistic BET-HP treatment (CTMP3-BET-HP). The synergy between integrated aqueous betulin surface engineering and hot-pressing is evident in the filter paper sample, where the wet strength index increases from nearly 0 to 4.9 kNm/kg (filter paper-BET-HP). Just hot-pressing filter paper alone results in an increase of 3.1 kNm/kg (filter paper-HP). Thus, it is the densification of the cellulose fibers, synergistically acting with the polymorphic transformation of the betulin crystals during heating, that contributes to the additional increase in the wet strength. The SEM and XRD analyses enable us to propose a mechanism for the synergistic betulin/hot-pressing technology. During hot-pressing, the fibers undergo densification, while simultaneously, betulin undergoes a polymorphic transformation into long whiskers. These whiskers interconnect through the cellulose fiber network, acting as a reinforcement/armature within the newly formed structure. In the case of lignocellulosic fibers, an additional synergistic effect occurs when chemical modification via sulfonation lowers the *T*_g_ of lignin, facilitating strong inter-fiber bonding and “gluing” of the fibers together, coupled with the polymorphic transformation of betulin into long whiskers. The higher degree of sulfonation, when combined with aqueous betulin modification and hot-pressing engineering, results in the highest wet strength. Thus, chemical modification has a significant synergistic effect with both the colloidal betulin particle/water suspension treatment as well as the hot-pressing.

## Conclusion

In summary, an eco-friendly, high-yielding, industrially relevant, and scalable method inspired by birch bark for fabricating hydrophobic and strong cellulosic materials is disclosed. The method combines surface modification of cellulosic fibers with colloidal betulin particles in water with sustainable engineering (e.g. hot-pressing and lignin modification). Thus, water-based formulations of betulin were developed for eco-friendly surface modification of the cellulosic fibers and next integrated with hot-pressing. This transformative process altered the morphology of the resulting hydrophobic cellulosic materials, in which the dense compact structure with betulin particles underwent polymorphic transformations from prismatic crystals (betulin III) to orthorhombic whiskers (betulin I). Significant synergistic effects were observed, resulting in a remarkable increase in wet strength of the fabricated hydrophobic cellulosic materials (contact angles of up to >130°). Thus, the presented scalable and synergistic approach holds great potential for advancing the development and application of high-performance cellulosic materials using renewable natural resources and sustainable chemistry. In this context, the concept is within the principles of green chemistry by demonstrating a novel way of synergistic and environmentally benign hydrophobization of cellulosic materials, which avoids the use of toxic forever chemicals and replaces them with a natural, readily available, and healthy natural compound. The synergistic betulin approach notably also omits the use of toxic and synthetic wet-strengthening agents such as epichlorohydrin. In fact, the improvement of strength in the fabricated hydrophobic cellulosic materials, which can be very high yielding (>95%) starting from soft wood, reduces the amount of lignocellulose needed for reaching a specific strength. This has tremendous importance in reducing the volume of starting trees needed for cellulosic material production.

## Supporting Information Available

Instruments and measurements; pulp additional information; additional procedures and results; SEM, size distribution, optical-microscopy image, and XRD spectra of betulin; FTIR spectrum and SEM images of the samples; WCA photographs; NMR spectra (PDF)

## Abbreviations

CTMP: chemi-thermomechanical pulp
FTIR: Fourier-transform infrared
NMR: nuclear magnetic resonance
DLS: dynamic light scattering
SEM: scanning electron microscope
XRD: X-ray diffraction
WCA: water contact angle
