# Supplementary material for: Birch-Bark-Inspired Synergistic Fabrication of High-Performance Cellulosic Materials
Source: ACS Sustain Resour Manag. 2024 Nov 13;1(12):2554–63. doi: 10.1021/acssusresmgt.4c00266 (PMC11684174; doi:10.1021/acssusresmgt.4c00266)
Supplement: Supplementary file 1 — rm4c00266_si_001.pdf [file rm4c00266_si_001.pdf]

Birch-bark Inspired Synergistic Fabrication of High-Performance Cellulosic Materials

Abdolrahim A. Rafi,[†] *Luca Deiana,*[†] *Rana Alimohammadzadeh,*[†] *Per Engstrand,*[‡] *Thomas Granfeldt,*[‡] *Staffan K. Nyström*[‡] *& Armando Cordova*[†*]

[†] FSCN Research Center, Organic Chemistry, Mid Sweden University, Holmgatan 10, 851 70, Sundsvall, Sweden. [*]E-mail: armando.cordova@miun.se

[‡] FSCN Research Center, High Yield Pulp Technology, Mid Sweden University, Holmgatan 10, 851 70, Sundsvall, Sweden.

Table of Contents

Materials 3
Pulp additional information 3
Sulfur total content 3
Wet and dry tensile tests 3
Fourier transform infrared spectroscopy (FT-IR) 3
Scanning Electron Microscope (SEM) images 4
NMR spectra 4
Optical microscopy 4
Dynamic light scattering 4
Hot-Pressing instrument 4
Water contact angle measurements 4
Typical procedure for the treatment of cellulosic materials with betulin/EtOH solution. 5
Preparation of betulin/EtOH solution (8.5 mM). 5
Treatment of cellulosic materials with betulin/EtOH solution (8.5 mM). 5
Table S1. Water contact angle measurements for the treated cellulosic materials. 5
Treatment of cellulosic materials with betulin solution in the presence of citric acid 5
Aqueous betulin formulation development for the treatment of cellulosic materials. 5
Preparation of betulin aqueous suspension 5
Figure S1.SEM of starting betulin powder, and freeze-dried betulin aqueous suspension 6
Figure S2. Optical microscopy images of the prepared betulin aqueous suspension 6
Figure S3. Particle size distribution of the betulin aqueous suspension. 7
Figure S4. XRD spectra of starting betulin powder, betulin aqueous suspension (dried at 55°C) and betulin aqueous suspension (freeze-dried). 7
Figure S5. The prepared betulin aqueous suspension 8
Impregnation treatment of cellulosic materials with betulin aqueous suspension and heat drying 8
Table S2. Water contact angle for different cellulosic-BET materials 8
Spraying treatment of cellulosic materials with betulin aqueous suspension and hot pressing: 9
Table S3. Betulin content of the cellulosic materials sprayed with betulin aqueous suspension before hot press. 9
Figure S6. Photographs of the samples 9
Figure S7. FTIR spectrum of CTMP2 samples 10
Figure S8. FTIR spectrum of CTMP3 samples 11
Figure S9. FTIR spectrum of filter paper samples 12
Figure S10. SEM images of filter paper samples. 13
Figure S11. SEM images of CTMP2 samples 13
Figure S12. SEM images of CTMP3 samples 14
Figure S13. The SEM images of CTMP1 samples after immersing in water 15
Figure S14. Photographs of water contact angles of samples 16
Figure S15. ^{1}H-NMR spectra of betulin. 16
Figure S16. ^{1}H-NMR spectra of melted betulin after heating at 280ºC 17
Figure S17. ^{13}C-NMR spectra of betulin. 17
Figure S18. ^{13}C-NMR spectra of melted betulin after heating at 280ºC 18
Figure S19. Planar hot-pressing instrument at Mid Sweden University 18
Table S4. Data from wet and dry tensile tests. 19
References 20

General information

Materials

Betulin (98%) was obtained from Holmen AB and Nature Science Technologies Ltd. Filter paper (No. 0B, diameter 18.5 cm) was obtained from Munktell. The raw material for pulping consisted of Norway spruce chips (*Picea abies*), of which 30 % was sawmill chips, from Billerud Rockhammar mill. The pulp is from the mill trials in Rockhammar 20-22nd February 2019. The samples are all CTMP with different amounts of bound sulphur (S). CTMP1 (total bound S of 2.4 g (S)/kg), and CTMP2 (total bound S of 1.0 g (S)/kg) with 28% dryness are from the primary high consistency refiner. CTMP3 with 85% dryness is form final step and the same as CTMP2 (total bound S of 1.0 g (S)/kg).

Pulp additional information

Steamed chips were impregnated with bisulphite and sodium hydroxide at initial pH 8.0. The residence time in the impregnator and the reaction vessel was typically 30 min and the temperature was about 65 °C. After that, the chips were preheated to about 145 °C and then fed into a pressurized 68-inch single disk refiner RGP 268 (Valmet). Pulp samples were collected from the blow line via a custom-made sampler. The time from preheating to sampling, was approximately 6 min. Extra bisulfite and sodium hydroxide was added to the refiner inlet. The residence time in the refiner was calculated to 1.5 sec. Composite pulp samples were collected during a three-minute period and thereafter homogenized by hand. Any excess air in the bags was removed and the bags were immediately sealed. To minimize chemical reactions (sulfonation) after sampling the bags were therefore stored at 5 °C overnight and then frozen and kept at −18 °C until the pulps were analyzed [1]. CTMP1 (with bound sulfur of 2.4 (S) g/kg; refining peak temperature of 165 °C; specific refining energy of 788 kWh/t; refining gap of 0.57 mm), and CTMP2 (with bound sulfur of 1.0 (S) g/kg; refining peak temperature of 165 °C; specific refining energy of 809 kWh/t; refining gap of 0.55 mm) are from primary high consistency refiner. CTMP3 with 85% dryness is form final step and the same as CTMP2 (bound sulfur content of 1.0 (S) g/kg) [1].

Instruments and measurements

Sulfur (S) total content

The total content of sulfur in the pulp samples were measured at MoRe Research in Örnsköldsvik, Sweden (SCAN-CM 57 method).

Wet and dry tensile tests

The tests were performed after conditioning in the standard testing environment according to in ISO 187 (23 °C, and 50 % relative humidity). Grammage, density, and thickness were determined according to ISO 536 and ISO 534 and ISO 5270, respectively. Dry tensile strengths and wet tensile strengths (after immersion in DI water for 1 min) were determined according to ISO 1924 and ISO 3781, respectively.

Fourier transform infrared spectroscopy (FT-IR)

FT-IR spectra were recorded using Thermo Scientific NICOLET 6700 FT-IR (Smart orbit, Diamond 30,000–200 cm^{-1}).

Scanning Electron Microscope (SEM) images

The SEM images were recorded using FE-SEM Tescan MAIA3. The samples were sputtered with 5 nm Ir layer using a Quorum Q150T sputter coater before imaging. The cross-section cutting was performed by an argon ion milling (Hitachi IM4000Plus).

NMR spectra

^{1}H NMR spectra were recorded on a Bruker Avance 500 (500 MHz) spectrometer. Chemical shifts are reported in ppm from tetramethylsilane with the solvent resonance resulting from incomplete deuterium incorporation as the internal standard ($CDCl_3$: δ 7.26 ppm). Data are reported as follows: chemical shift, multiplicity (s = singlet, d = doublet, q = quartet, br = broad, m = multiplet), and coupling constants (Hz), integration. ^{13}C NMR spectra were recorded on a Bruker Avance 500 (125.8 MHz) spectrometer with complete proton decoupling. Chemical shifts are reported in ppm from tetramethylsilane with the solvent resonance as the internal standard ($CDCl_3$: δ 77.26 ppm).

Optical microscopy

For optical microscopy, a drop of sample was put on a glass slide and covered with a coverslip. The sample was observed using a microscope (Leitz Wetzlar) equipped with a microscope lens adapter (FMA050, Toupcam™) and a digital camera (U3CMOS, Toupcam™).

Dynamic light scattering

Dynamic light scattering instrument (Zetasizer Nano-ZSP, Malvern Instruments) was used to measure the average particle size and size distribution. The prepared betulin/water suspension was diluted to 200 µg/ml and the measurement was performed at 25 °C in triplicate.

Hot-Pressing instrument

Hot pressing was performed via a planar-pressing equipment built at Mid Sweden University, and the equipment mainly consists of heating blocks, pillar stand, and compression testing machine (figure S19). The heating blocks contain three pockets where electrical heating elements (each 500 W) are located. The heating blocks, with 20 mm thermal isolating plates, are mounted in pillar rack ball brushings to ensure the best alignment between the blocks. The upper pillar rack is fixed to a hydraulic MTS™ material testing machine loadcell and the lower part to the movable hydraulic piston rod. For the control of block temperature, each has a built-in thermocouple sensor connected to Eurotherm PID-type regulators which are limited to 300 °C. To control compression loads, MTS™ MPT software is used for creating block-programmed load vs. time sequences up to 100 kN.

Water contact angle measurements

The static WCA measurements were recorded on a PGX+ Pocket Goniometer. The static contact angles were measured after 5 min and after placing a 4 µL Milli-Q water droplet onto the sample surfaces and an average of 5 measurements was reported for each sample.

X-Ray Diffraction (XRD) spectra

The XRD spectra were acquired using “Bruker AXS D2 Phaser”. The X-ray generator was equipped with a Cu tube operating at 30 kV and 10 mA and irradiating the sample with a monochromatic CuKα radiation with a wavelength of 1.54 Å. XRD spectra were recorded at room temperature over the 2-theta range of 5°–70° with sampling at 0.02° increments and with a measurement time of 1 s per 2θ intervals.

Betulin powder samples from betulin aqueous suspension were obtained by drying of betulin aqueous suspension at oven (55 °C), and also freeze drying of betulin aqueous suspension (-110 °C, 0.012 hPa). To prepare CTMP1-BET-NoHP for XRD analysis, CTMP1-starting was sprayed with the prepared betulin aqueous suspension and then dried at 55 °C (spraying and drying was repeated for 3 times). Hot pressing of the prepared CTMP1-BET-NoHP (250 °C, 3 bar, 60 s) produced CTMP1-BET-HP sample.

Typical procedure for the treatment of cellulosic materials with betulin/EtOH solution.

Preparation of betulin/EtOH solution (8.5 mM).

Ethanol (100 mL) was added to betulin (375 mg, 0.85 mmol) and the solution was stirred until completely solubilization of betulin.

Treatment of cellulosic materials with betulin/EtOH solution (8.5 mM).

The cellulosic material was impregnated with a betulin/EtOH solution (8.5 mM) for the time reported in the table and then air dried. Betulin appears as a not evenly spread powder on the cellulosic material surface. Table S1 shows the impregnation time and contact angles for the treated cellulosic materials.

Table S1. Water contact angle (WCA) measurements for the treated cellulosic materials.

Entry	Cellulosic material	Impregnation time	WCAs (θ)[a]
1[b]	CNC film	0	45°
2	CNC film	10 min	80 °
3	CNC film	4 h	83 °
4	CNC film	18 h	82 °
5	Filter paper	3 h	< 10 °
6[c]	Filter paper	3 h	< 10 °

[a] The contact angle was measured after 10 seconds. [b] Not impregnated with the betulin solution. [c] Hot pressed at 97°C, 1 bar for 30 minutes.

Procedure for the treatment of cellulosic materials with betulin solution in the presence of citric acid.

In a round bottom flask was added betulin (141 mg, 0.32mmol), THF (40 mL), citric acid (61 mg, 0.32 mmol) and filter paper (57 mg, 0.32 mmol glucose). The solution containing the filter paper was slowly stirred at 70°C for 21 hours. Next, the filter paper was removed from the solution and air dried to measure the contact angle (Contact angle: < 10°).

Aqueous betulin formulation development for the treatment of cellulosic materials.

Preparation of betulin aqueous suspension (20 mg/mL).

Deionized water (300 mL) was slowly added to betulin (6 g, 13,55 mmol), and vigorously stirred at room temperature for 1 h. Next, the suspension was sonicated at 35 °C for 1.5 h to give a homogeneous milky solution.

SEM images of starting betulin powder, and freeze-dried betulin aqueous suspension, as well as optical microscopy images of the prepared betulin aqueous suspension are shown in Figure S1 and S2, respectively.

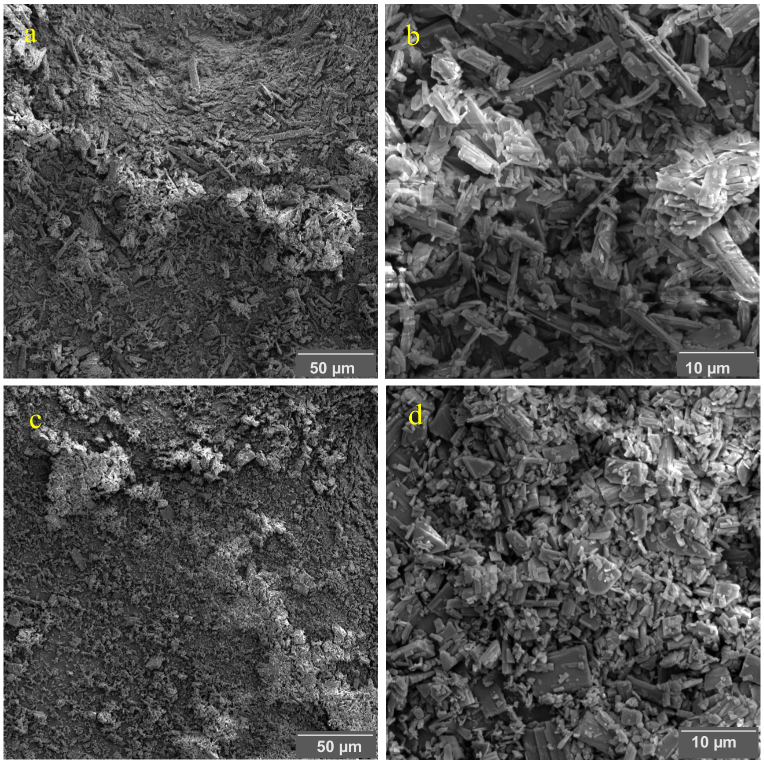

Figure S1. SEM of starting betulin powder (a,b), and freeze-dried betulin aqueous suspension (c,d).

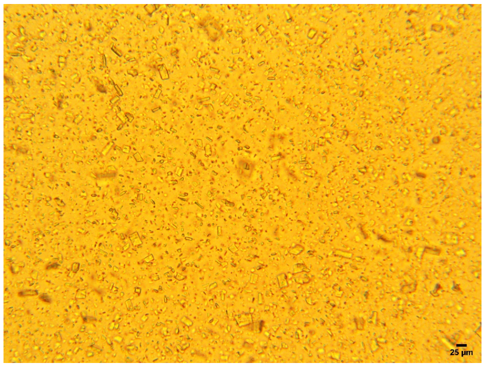

Figure S2. Optical microscopy images of the prepared betulin aqueous suspension (scale bar: 25µm).

The DLS measurement of betulin aqueous suspension (200 µg/mL) showed the average particle size of around 857±16 nm and polydispersity index (PDI) of 0.31±0.06.

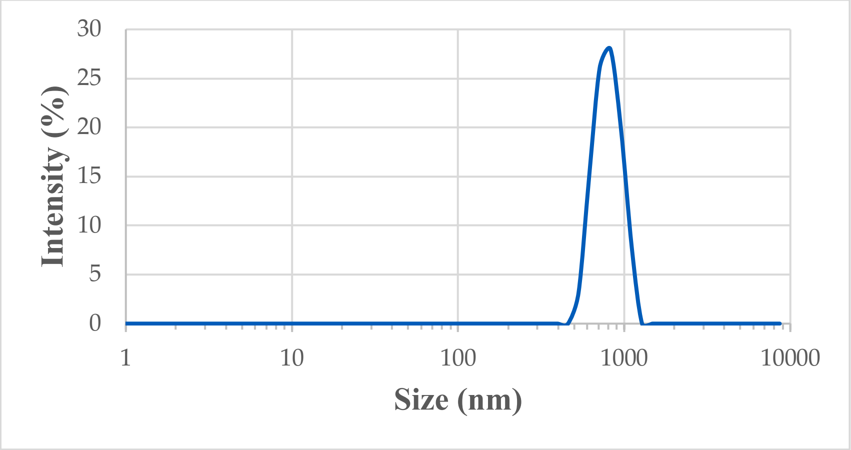

Figure S3. Particle size distribution of the betulin aqueous suspension.

Figure S4 depicts the XRD spectra of the starting betulin powder, betulin after the aqueous suspension was dried at 55°C and betulin after the aqueous suspension was freeze-dried. The XRD spectra of all these samples shows that they have the same polymorph, which is betulin hydrate (betulin III).

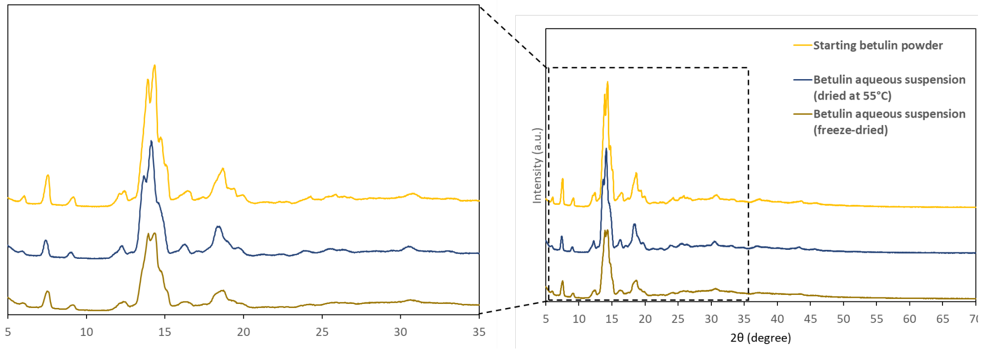

Figure S4. XRD spectra of starting betulin powder, betulin aqueous suspension (dried at 55°C) and betulin aqueous suspension (freeze-dried).

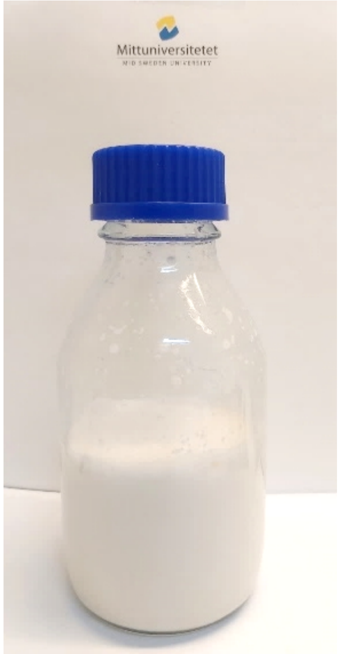

Figure S5. The prepared betulin aqueous suspension (20 mg/mL).

Impregnation treatment of cellulosic materials with betulin aqueous suspension (20 mg/mL) and then heat drying:

The cellulosic material was impregnated with the betulin aqueous suspension (20 mg/mL) for 10 minutes. Next, the cellulosic material was removed from the suspension and left to air dry. The corresponding betulin impregnated cellulosic materials were next heated in a furnace for 3 minutes at 280 ºC. Table S2 shows the contact angle of corresponding cellulosic-BET samples.

Table S2. Water contact angle for different cellulosic-BET materials

Entry	Cellulosic material	Contact angle (θ)[a]
1	Filter paper-BET	110º
2	CTMP3-BET	118º
3	CTMP2-BET	114 º
4	CTMP1-BET	120 º
5	CNC coated paper-BET	83 º
6	Cotton fabric-BET	130 º
7[b]	Filter paper-BET	nd
8[c]	Filter paper-BET	< 10º

[a] The contact angle was measured after 5 min. [b] Sodium dodecyl sulfate SDS (20 mol%) was tested as surfactant in the betulin/H_2O suspension. SDS burn at 280 ºC. [c] Pluronic P-123 (1.5 mol%) was tested as surfactant in the betulin/H_2O suspension. Nd= not determined.

Spraying treatment of cellulosic materials with betulin aqueous suspension (20 mg/mL) and hot pressing:

The prepared CTMP handmade sheets and filter paper samples were sprayed with the betulin aqueous suspension and successively dried in oven at 55 °C for 1 h (BET-NoHP samples, table S3). Finally, samples were hot pressed at 3 bar and 250 °C for 1 min, giving BET-HP samples (Figure S6).

Table S3. Betulin content of the cellulosic materials sprayed with betulin aqueous suspension before hot press.

Cellulosic Material	Starting weight[a] (g)	Coated weight[b] (g)	Betulin Content (g)	Betulin percentage (%)
Filter paper	2.477 ± 0.015	2.623 ± 0.025	0.147 ± 0.012	5.6
CTMP1	4.150 ± 0.020	4.407 ± 0.030	0.257± 0.012	5.8
CTMP2	4.210 ± 0.014	4.460± 0.028	0.250± 0.014	5.6
CTMP3	4.285 ± 0.035	4.555 ± 0.035	0.270 ± 0.004	5.9

[a] Weight of the cellulosic material after 24 hours in the conditioning room at 23°C and 50% relative humidity. [b] Weight of the betulin coated cellulosic material after drying in the oven and kept for 24 hours in the conditioning room.

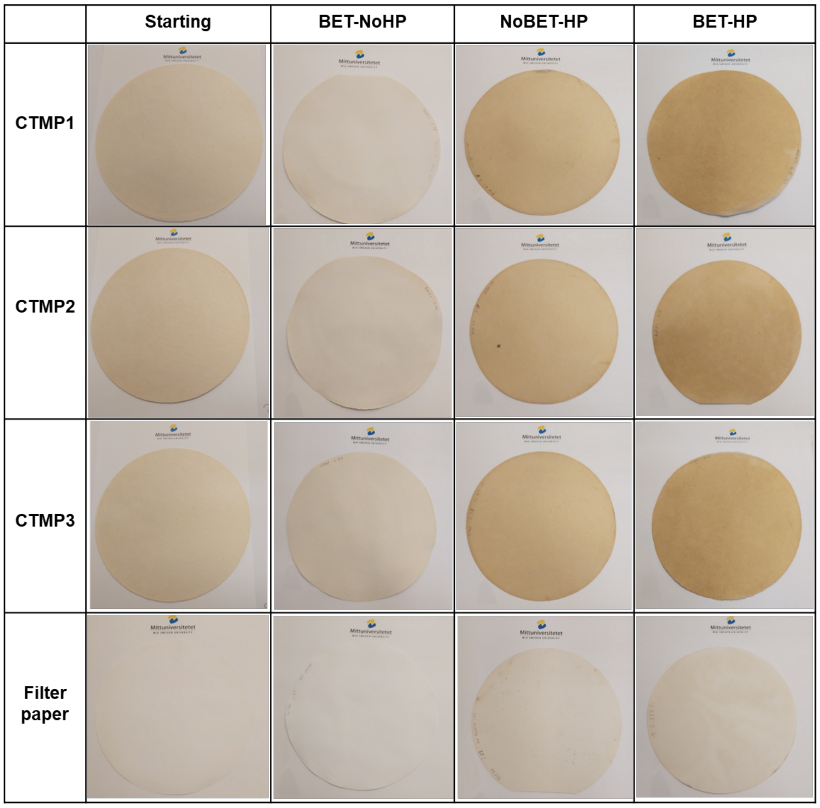

Figure S6. Images of filter paper, and all CTMP samples without betulin and without hot pressing (starting), with betulin without hot pressing (BET-NoHP), with betulin without hot pressing (NoBET-HP) and with betulin and with hot pressing (BET-HP).

FTIR spectra of CTMP2, CTMP3, and filter paper samples are shown in Figure S7-S9, respectively.

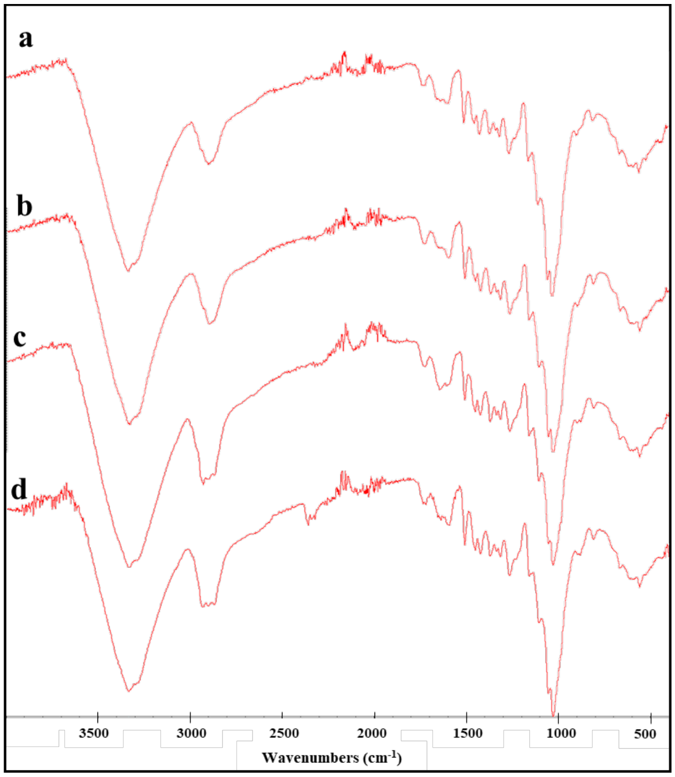

Figure S7. FTIR spectrum of CTMP2-starting (a), CTMP2-NoBET-HP (b), CTMP2-BET-NoHP (c), CTMP2-BET-HP (d).

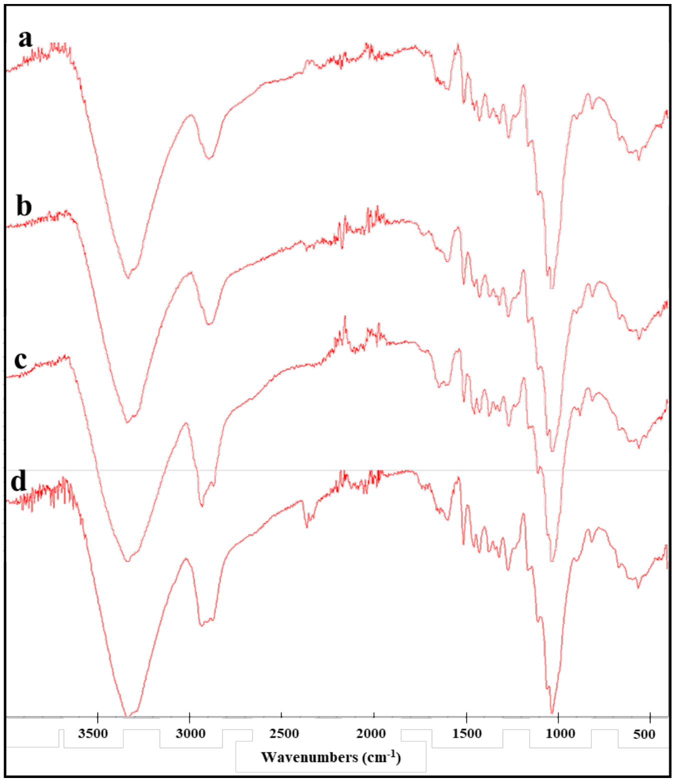

Figure S8. FTIR spectrum of CTMP3-starting (a), CTMP3-NoBET-HP (b), CTMP3-BET-NoHP (c), CTMP3-BET-HP (d).

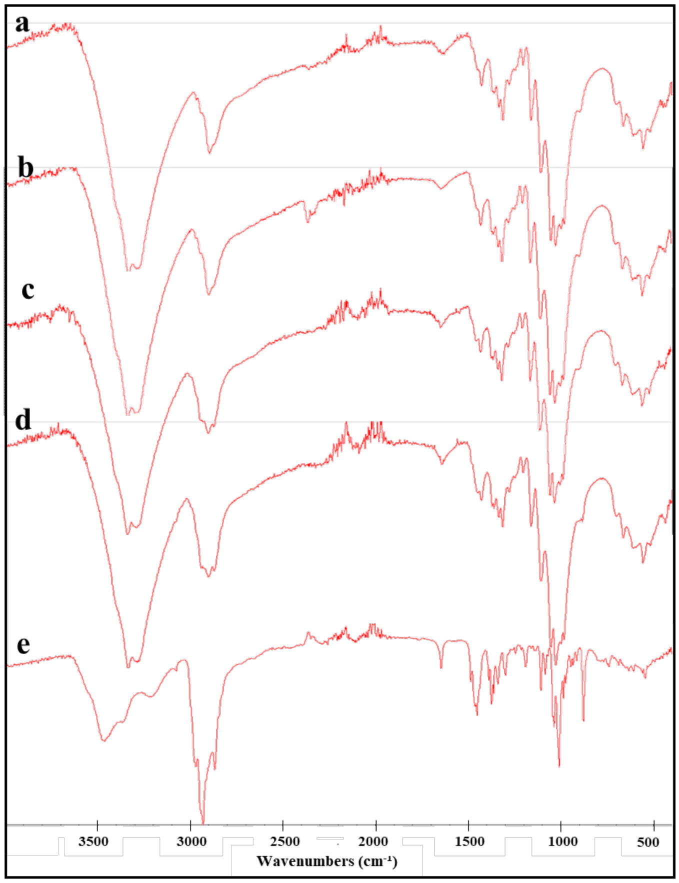

Figure S9. FTIR spectrum of filter paper-starting (a), filter paper-NoBET-HP (b), filter paper-BET-NoHP (c), filter paper-BET-HP (d), and betulin (e).

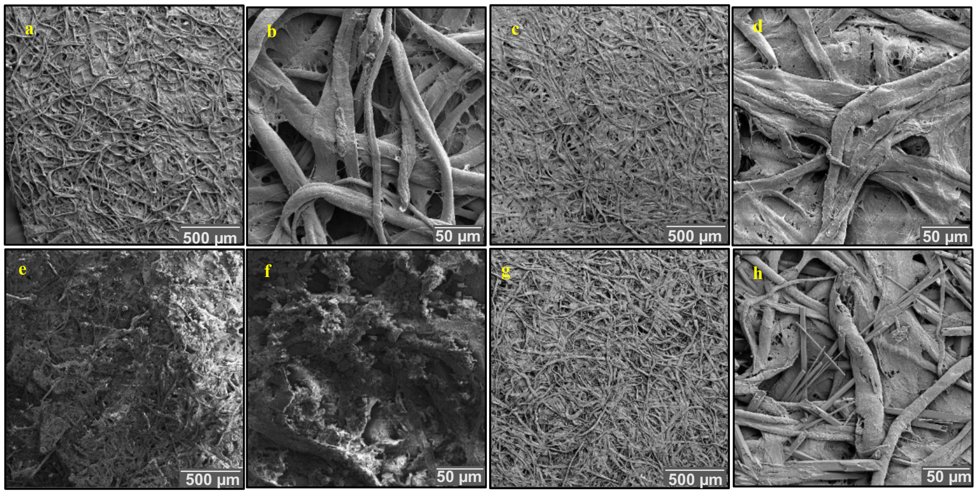

Figure S10. SEM images of filter paper-starting (a,b), filter paper-NoBET-HP (c,d), filter paper-BET-NoHP (e,f), filter paper-BET-HP (g,h).

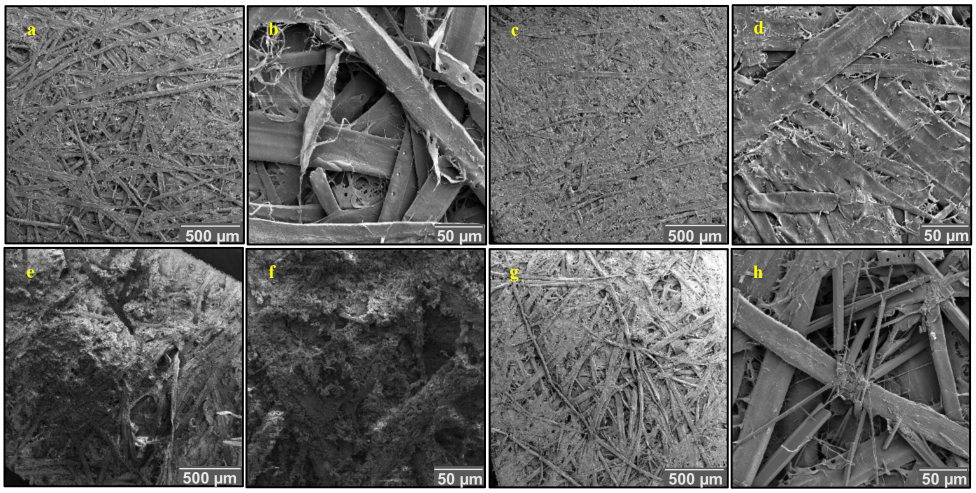

Figure S11. SEM images of CTMP2-starting (a,b), CTMP2-NoBET-HP (c,d), CTMP2-BET-NoHP (e,f), CTMP2-BET-HP (g,h).

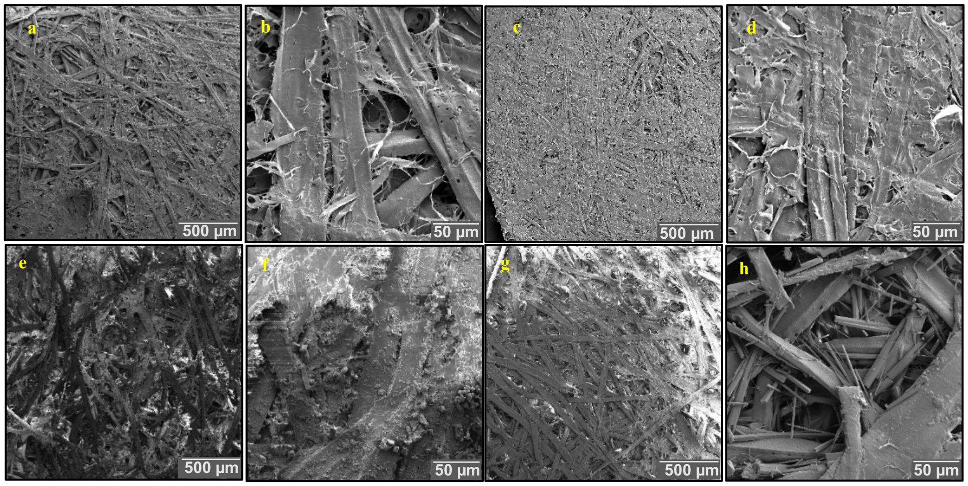

Figure S12. SEM images of CTMP3-starting (a,b), CTMP3-NoBET-HP (c,d), CTMP3-BET-NoHP (e,f), CTMP3-BET-HP (g,h).

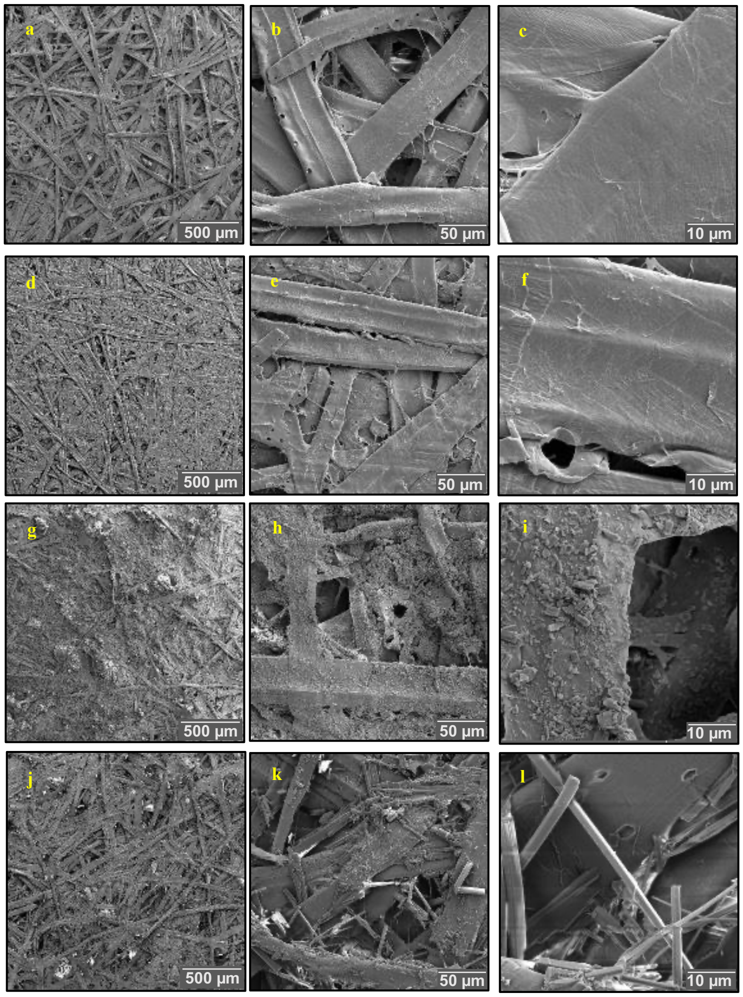

Figure S13. The SEM images of CTMP1-starting (a, b, c), CTMP1-NoBET-HP (d, e, f), CTMP1-BET-NoHP (g, h, i), and CTMP1-BET-HP (j, k, l) after immersing in deionized water (for 1 min) and air dried.

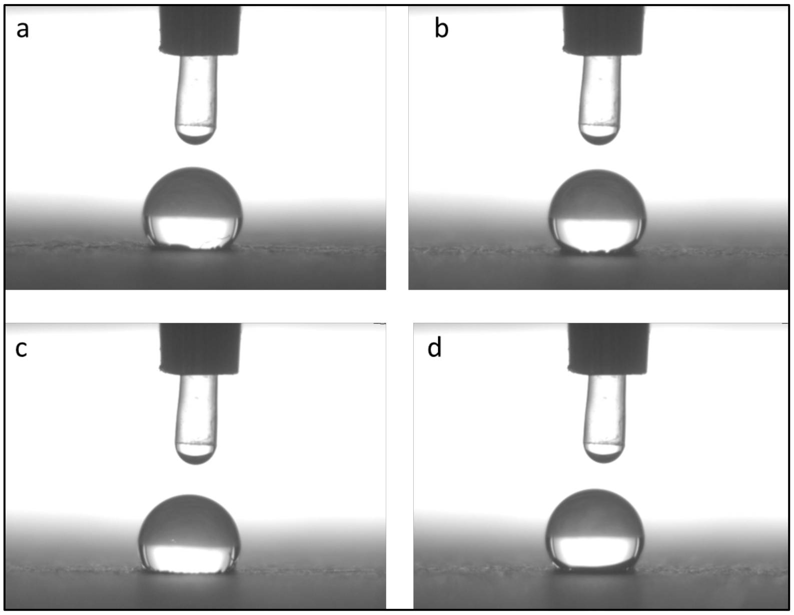

Figure S14. Water contact angle images of filter paper-BET-HP (a), CTMP3-BET-HP (b), CTMP2-BET-HP (c), and CTMP1-BET-HP (d) after 5 min.

^{1}H-NMR and ^{13}C-NMR spectra of betulin before and after heating at 280 °C are shown in Figure S15-S18.

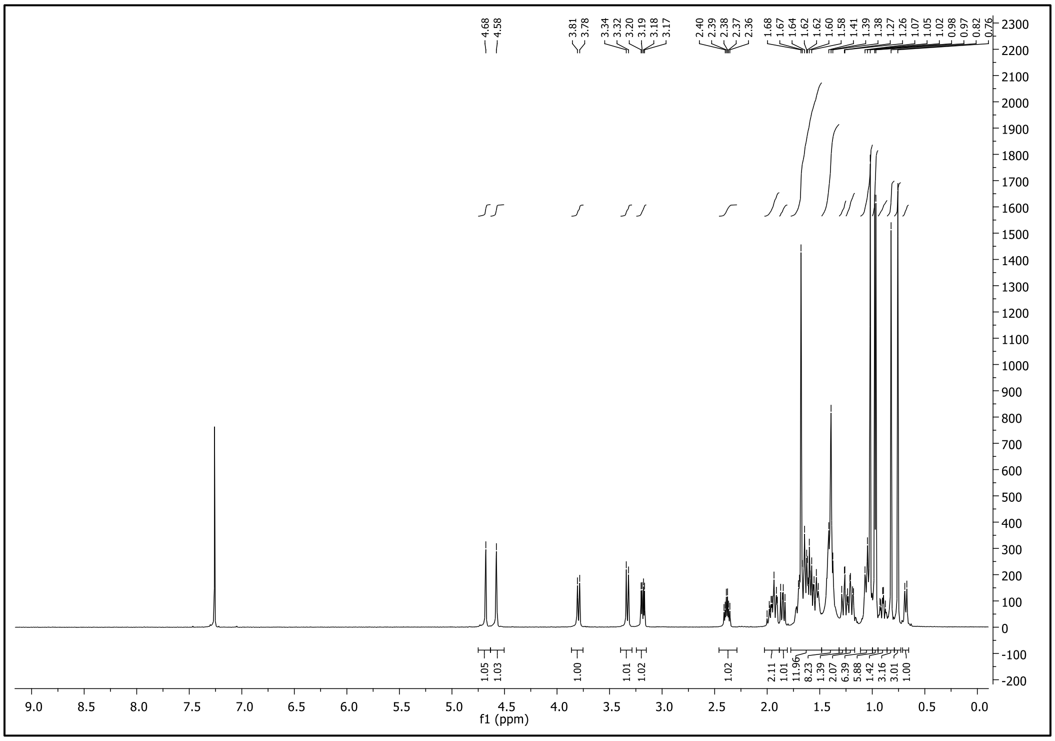

Figure S15. ^{1}H-NMR spectra of betulin.

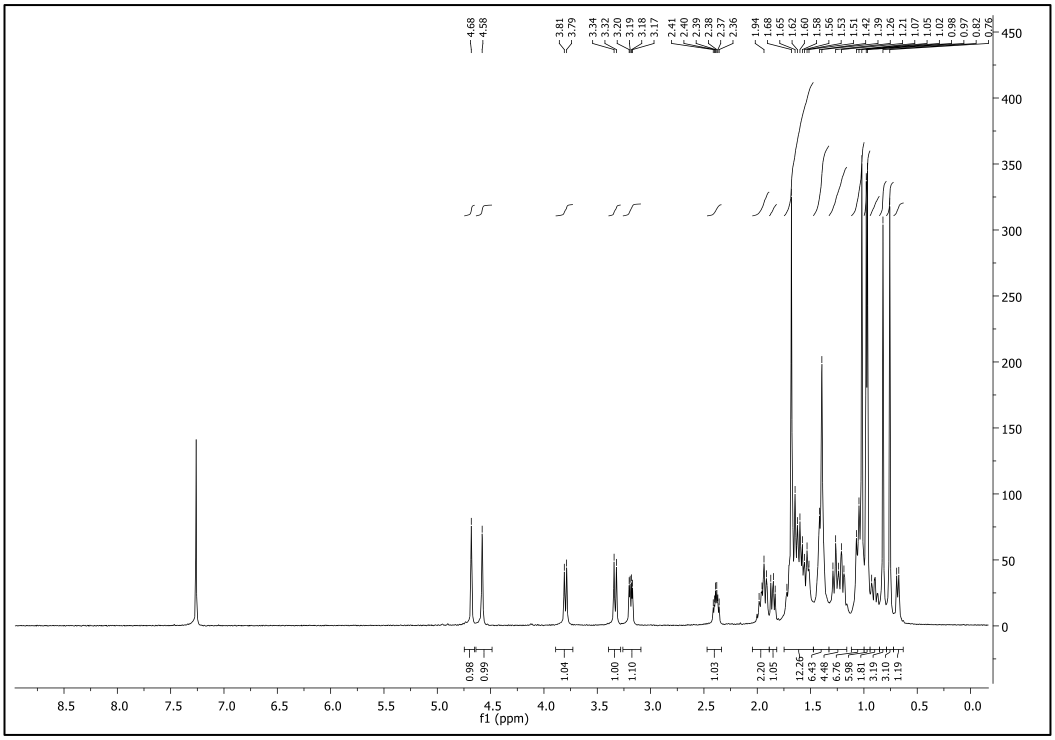

Figure S16. ^{1}H-NMR spectra of melted betulin after heating at 280°C.

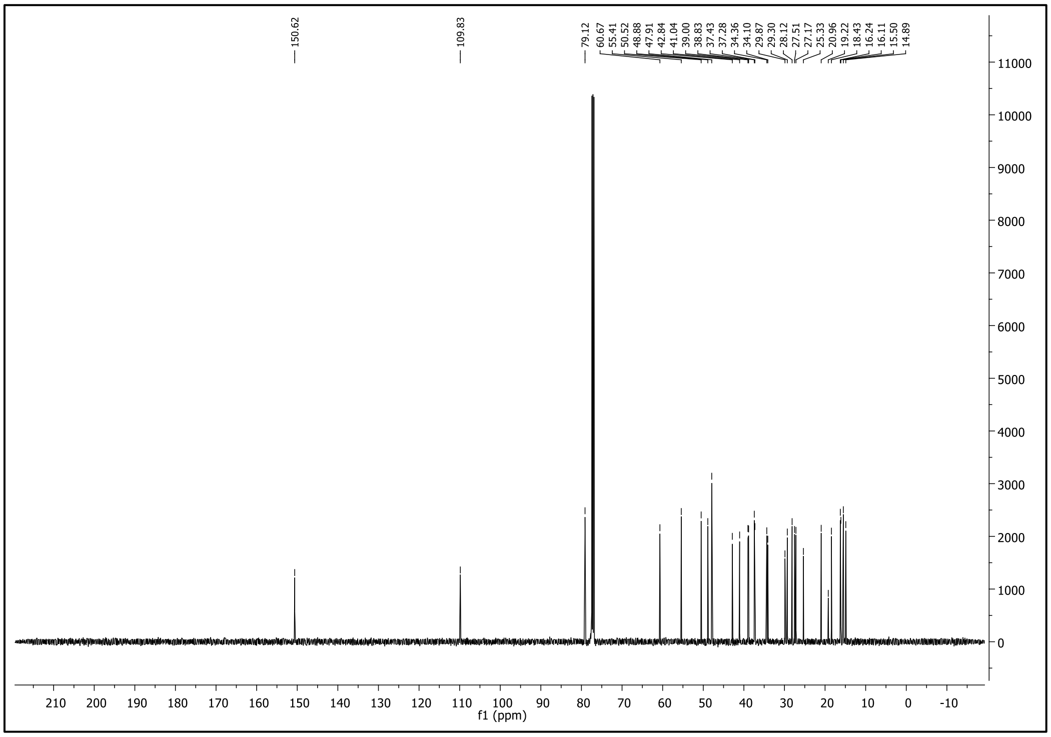

Figure S17. ^{13}C-NMR spectra of betulin.

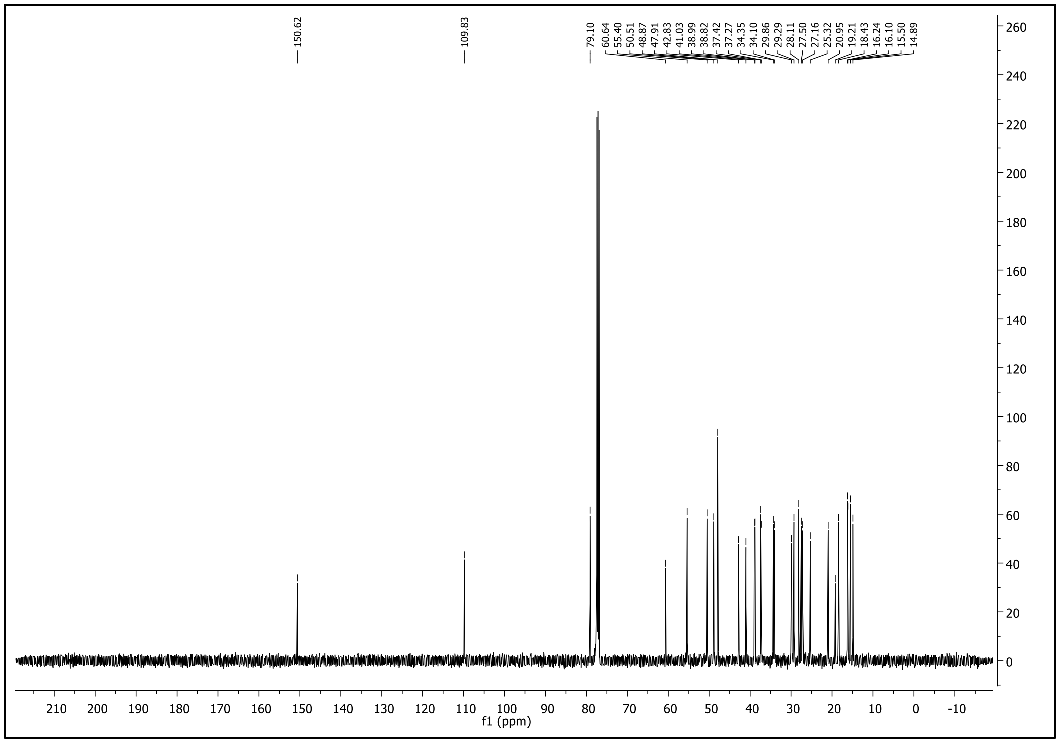

Figure S18. ^{13}C-NMR spectra of melted betulin after heating at 280ºC.

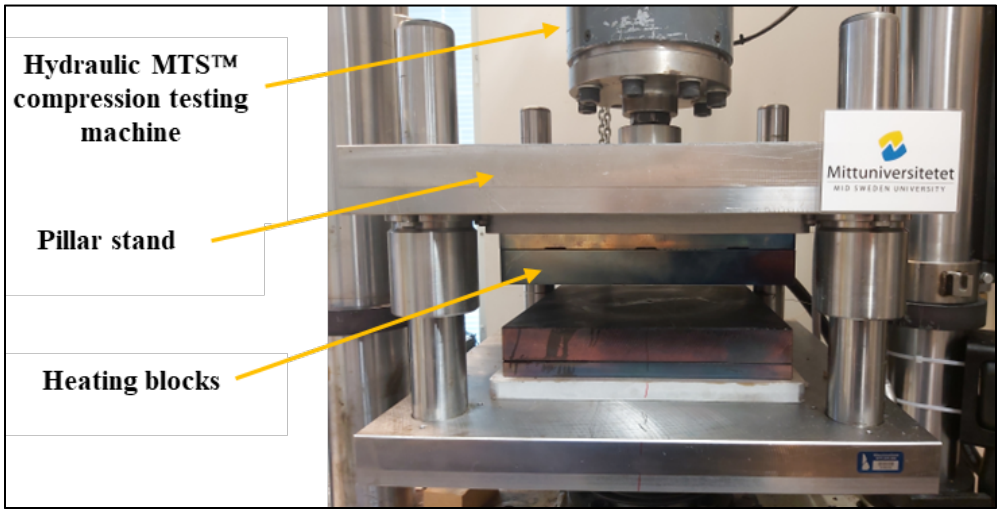

Figure S19. Planar hot-pressing instrument at Mid Sweden University.

Table S4. Data from wet and dry tensile tests.

	Filter paper				CTMP3				CTMP2				CTMP1			
	Starting	BET-NoHP	NoBET-HP	BET-HP	Starting	BET-NoHP	NoBET-HP	BET-HP	Starting	BET-NoHP	NoBET-HP	BET-HP	Starting	BET-NoHP	NoBET-HP	BET-HP
**Grammage (g/m^2)**	92	96	96	96	141	149	134	141	139	143	132	137	140	139	129	136
**Thickness (um)**	224	222	144	142	338	500	169	171	303	385	161	161	312	394	162	159
**Standard deviation**	4,0	14,8	5,1	2,5	8,1	19,5	8,4	1,2	11,0	9,4	5,4	3,0	8,8	13,5	6,1	1,9
**Density (kg/m^3)**	413	432	663	672	417	299	797	822	458	371	819	854	448	352	794	852
**Tensile strength (kN/m)**	1,1	1,5	1,3	1,6	4,0	2,0	5,3	4,9	5,2	3,6	6,7	5,9	5,6	3,5	6,4	6,3
**Standard deviation**	0,05	0,06	0,03	0,16	0,15	0,15	0,23	0,33	0,15	0,06	0,23	0,28	0,13	0,22	0,38	0,23
**Tensile index (kNm/kg)**	12,1	15,7	13,4	16,8	28,2	13,3	39,5	34,6	37,3	25,5	50,7	42,7	39,7	25,1	49,8	46,6
**Standard deviation**	0,5	0,6	0,3	1,7	1,1	1,0	1,7	2,4	1,1	0,4	1,7	2,1	0,9	1,6	2,9	1,7
**Tensile Stiffness (kN/m)**	166,4	226,3	206,9	293,4	652,6	300,4	763,4	997,6	740,1	446,1	845,5	985,4	733,9	425,8	817,8	1029,2
**Standard deviation**	8,8	16,8	6,7	31,3	26,1	10,7	39,5	43,7	23,9	15,1	20,3	23,1	24,0	21,8	13,1	32,3
**Tensile Stiffness Index (MNm/kg)**	1,8	2,4	2,2	3,1	4,6	2,0	5,7	7,1	5,3	3,1	6,4	7,2	5,2	3,1	6,3	7,6
**Strain at break (%)**	2,1	1,9	1,6	1,3	1,1	1,1	1,4	1,0	1,1	1,6	1,6	1,4	1,3	1,4	1,5	1,3
**Standard deviation**	0,16	0,1	0,1	0,19	0,12	0,15	0,06	0,13	0,13	0,09	0,10	0,15	0,03	0,12	0,14	0,27
**Wet-Tensile strength (kN/m)**	< 0,01	< 0,01	0,29	0,47	0,28	< 0,01	2,13	3,88	0,54	0,45	2,48	4,20	0,80	0,60	2,66	5,10
**Standard deviation**	_	_	0,01	0,04	0,01	_	0,11	0,27	0,03	0,01	0,17	0,24	0,02	0,11	0,03	0,43
**Wet-Tensile index (kNm/kg)**	_	_	3,1	4,9	2,0	_	15,8	26,9	3,9	3,2	18,8	30,7	5,7	4,3	20,7	37,6
**Standard deviation**	_	_	0,1	0,4	0,1	_	0,9	3,6	0,2	0,1	1,3	1,8	0,1	0,1	0,2	3,1
**Wet-tensile Stiffness (kN/m)**	_	_	40,1	66,8	56,6	_	177,7	461,4	81,2	66,1	186,8	418,2	85,8	64,2	179,0	497,3
**Standard deviation**	_	_	4,0	8,8	6,3	_	5,9	81,61	5,2	9,1	5,1	27,9	4,1	2,1	2,8	56,0
**Wet-tensile Stiffness Index (MNm/kg)**	_	_	0,4	0,7	0,4	_	1,3	3,3	0,6	0,5	1,4	3,1	0,6	0,5	1,4	3,7
**Strain at break (%)**	_	_	2,4	2,2	1,0	_	2,6	2,3	1,5	1,6	2,7	3,0	1,9	1,8	3,0	3,0
**Standard deviation**	_	_	0,20	0,34	0,09	_	0,20	0,3	0,07	0,05	0,31	0,26	0,10	0,10	0,08	0,33

References

1. Berg, J.-E. *et al*. Refining gentleness – a key to bulky CTMP. *Nord Pulp Paper Res J* **37**, 349–355 (2022).
